# The moderation effect of social capital in the relationship between own income, social comparisons and subjective well-being: Evidence from four international datasets

**DOI:** 10.1371/journal.pone.0288455

**Published:** 2023-12-07

**Authors:** Stefano Bartolini, Marcin Piekalkiewicz, Francesco Sarracino, Giulia Slater

**Affiliations:** 1 Department of Political Economy and Statistics, Faculty of Economics, University of Siena, Siena, Italy; 2 Institut national de la statistique et des études économiques du Grand-Duché du Luxembourg (STATEC), Luxembourg City, G.D. of Luxembourg; Universidad de la Republica, Facultad de Ciencias Sociales, URUGUAY

## Abstract

In this paper we check whether social capital changes the association of subjective well-being with own income and social comparisons. We use panel data from Germany and publicly available data from three international surveys, for a total of nearly 500,000 respondents from industrial countries. Results show that the association of own income and social comparisons to subjective well-being weakens for individuals with high social capital. This finding holds in a variety of settings, and is robust to various measures of subjective well-being, of social capital, and of social comparisons. We also find evidence indicating that the role of social capital is, at least in part, causal. Finally, our findings support the macro-level implication that income differences are less related to subjective well-being differences in countries with high social capital.

## 1 Introduction

In this paper, we study how social capital changes the relationship between income and subjective well-being, at both individual and aggregate level. We focus especially on the moderating role of social capital in the relationship between subjective well-being and income, considered both as a mean to satisfy individual’s needs (own income), and as a source of social comparisons.

Own income correlates positively with subjective well-being in micro-data because greater purchasing power increases individuals’ ability to satisfy their needs [1–3].

The correlation between income and subjective well-being is ambiguous when it is used as a mean to establish social comparisons. Social comparisons refer to people’s tendency to compare their own achievements with those of self-relevant others—those who form the so-called reference group. In present work, we refer to social comparisons as an umbrella term encompassing various measures of individual comparisons with others, such as reference income, self-reported social class and own income rank in the national income distribution. A long standing tradition in economics emphasizes the importance of relative economic position, beyond the absolute one. Veblen, one of the pioneers of this literature, emphasized the pursuit of status through consumption (which he called “conspicuous consumption”) [4].

Several studies documented a negative correlation between the income of others and subjective well-being [5–10]. Some studies, however, also found evidence of positive spillovers [11–13]. This can happen because the higher income of others induces a signalling effect: people are happier because the higher income of others signals an improvement that, sooner or later, will affect them as well [14]. Moreover, the scale of comparisons seem to matter in determining positive and negative spillovers [15]. Kingdon and Knight, in a study on South Africa, find negative spillovers of income at the district level (with average population size of 125,000 inhabitants), but positive spillovers within smaller communities (with average populations of 2,900 inhabitants) [16].

Independently from the sign, previous studies documented that own income and social comparisons matter for subjective well-being. However, there are reasons to believe that this relation depends on individual’s social capital. This aspect has gone largely unnoticed in the literature. Most studies take own income, social comparisons and social capital as mutually independent factors related to subjective well-being, with social capital playing a well-established positive role [17]. Researchers overlooked the possibility that social capital moderates the relationship of own income and social comparisons with subjective well-being. However, Veblen suggested that this might be the case. He argued that social comparisons grow as the cohesion between subjects decreases [4]. In his view, the more a community is composed of strangers—that is, the less social capital it has—the more conspicuous consumption becomes relevant.

This claim is echoed by findings from social psychology suggesting that social capital is negatively related to materialistic values. Materialistic individuals attach high importance in their life to both their absolute and relative economic achievements [18]. The psychological literature documented also that materialism is negatively correlated to social capital, suggesting that income and social status offer psychological compensation to the distress caused by poor social capital [19–21]. Social capital is commonly defined as “networks together with shared norms, values and understandings that facilitate cooperation within or among groups” [22] and it entails the formal and informal social relationships, the shared norms of reciprocity and trust within a community, as well as the emotional support, material and behavioral assistance between people [23, 24].

Psychological literature, however, analysed how social capital influences the importance of own income and social comparisons for individual values, but not for their well-being. What is more, studies are often based on small random samples that limit the possibility to draw general conclusions, and offer little evidence of a causal link between the variables. Pieters finds that materialism and loneliness are intertwined over time, with loneliness contributing to materialism more than the other way around in a longitudinal sample of individuals [25]. Moreover, experimental evidence suggests that materialism causes a reduction of helpfulness and generosity [26], and interest in relational activities [27], which are common measures of social capital.

We overcome these limitations by showing that social capital moderates the relationship of own income and social comparisons with subjective well-being in large and nationally representative data, and by providing some evidence on the causal relation among our variables of interest.

To the best of our knowledge, only Barcena-Martin and colleagues have tested the hypothesis that social capital moderates the relation between social comparisons and subjective well-being [28]. The authors used data from the German Socio-Economic Panel, and explored the hypothesis that two types of social capital, bridging and bonding, moderate the effects of relative income on subjective well-being. Bonding social capital concerns relationships among individuals belonging to a group or community, whereas bridging social capital refers to relationships among individuals belonging to different social groups (by social class, race, religion, etc.). The authors find that bridging social capital moderates the relationship between subjective well-being and social comparisons, while bonding social capital does not play a statistically significant role.

Our contribution is to widen the generality of previous conclusions. Specifically, we build on the work by Barcena-Martin and colleagues and extend its geographical scope; we check the robustness of their conclusion to a variety of measures; we account for the potential endogeneity of social capital, arguing that the moderating role of social capital is, at least in part, causal; importantly, we extend the analysis of the moderating role of social capital to own income, whereas Barcena-Martin and colleagues focus only on social comparisons. We use panel data from Germany and publicly available data from three international surveys, for a total of nearly 500,000 respondents from industrial countries.

In addition, we check the correlation between social capital at country level and a measure of well-being inequality among income groups, i.e. the subjective well-being difference between rich and poor people. If income is less strongly associated to the subjective well-being of individuals with high social capital, then in countries where social capital is high the well-being differences between rich and poor people should be smaller than in countries with low social capital.

Previous studies adopted various measures of social capital, such as the frequency of attending social activities, meetings with friends, relatives or neighbors, as well as volunteering, cooperative attitudes of people, and various measures of trust in others and in institutions [29–34]. In the present study, we use the data available at hand, which include measures of social contacts, interpersonal trust and individuals’ associational activity.

We find that own income and social comparisons are less strongly associated to subjective well-being in individuals with high social capital, than in others. We also find that subjective well-being differences between rich and poor people are smaller in countries with high social capital. In other words, the higher a country’s social capital, the less income inequality translates into subjective well-being inequality.

Our results differ from those by Barcena-Martin and collegues on one point. They found that bonding social capital does not moderate the impact of social comparisons on well-being, whereas our measures of social capital do. We use a different set of social capital variables to retain a larger sample than the one used by Barcena-Martin and colleagues, and, although we do not distinguish between bridging and bonding social capital, our measures of social capital include proxies of bonding social capital.

In the next two sections we present the data and the empirical method. In section 4, we illustrate the main results of the analysis. Section 5 reports the results of the endogeneity tests, whereas section 6 presents the results of the country-level analysis on the life satisfaction gap. In section 7 we discuss the implications of our findings for policy-making, while section 8 summarizes our findings and concludes.

## 2 Method

Checking how own income and social comparisons correlate with subjective well-being in correspondence with various levels of social capital requires estimating a standard happiness regression with interaction terms. To this purpose, we apply Ordinary Least Squares (OLS) regression analysis.

Eq 1 provides a general form of the equation we tested. The exact definition of the variables, as well as the list of control variables changes depending on the dataset. These aspects are presented in section 3.

$$\begin{array}{c} \begin{array}{cc} SWB_{i} & =\alpha +\beta_{1}\cdot Income_{i}+\beta_{2}\cdot SocComp_{i}+\beta_{3}\cdot SCindex_{i}+\\ & +\beta_{4}\cdot SCIndex_{i}\cdot Income_{i}+\beta_{5}\cdot SCindex_{i}\cdot SocComp_{i}+\\ & +\gamma^{\prime }X_{i}+\varepsilon_{i} \end{array} \end{array}$$ (1)

where *SWB* stands for subjective well-being, a general term encompassing various measures of reported well-being; the subscript *i* stands for individuals; *Income* and *SocComp* stand for one’s own income and social comparisons, respectively; *SCindex* is a categorical variable where higher values indicate a higher level of social capital; **X** is a vector of control variables such as age, gender, marital status, education level, a health variable, country and year dummies (when they apply). The full list of controls included is specified in the data section and in the notes to Table A2 in the S1 Appendix. Lastly, *ε* is the error term. All estimates make use of heteroskedasticity robust standard errors.

Interaction terms (*β*_4_ and *β*_5_) indicate whether the impacts of own income and social comparisons on subjective well-being change with the level of social capital. The marginal effects of own income and social comparisons on subjective well-being are then respectively equal to the expressions *β*_1_ + *β*_4_ ⋅ *SCindex*_*i*_ and *β*_2_ + *β*_5_⋅*SCindex*_*i*_.

For ease of interpretation of the results, and in particular of interaction effects, we estimate Eq 1 using OLS, thus treating subjective well-being as a cardinal variable. Our results, however, are qualitatively unchanged if we use ordered probit regressions. For the analysis of SOEP data, we modify Eq 1 to include individual fixed effects to account for time invariant unobserved heterogeneity. This is possible because of the longitudinal dimension of the data. Also in this case we use robust standard errors.

### 2.1 Moderation effects

We quantify the role of social capital by means of moderation effects which indicate by how much each level of social capital reduces the coefficients of own income and social comparisons in a subjective well-being regression.

The computation of moderation effects proceeds as follows:

1. the main effects (*β*_1_ and *β*_2_) from Eq 1 provide the baseline correlation between own income, social comparisons, and subjective well-being;
2. we add the interaction effects (*β*_4_, *s* and *β*_5_, *s*) to the main effects to compute the correlation of social comparisons with subjective well-being for the various levels *s* of social capital (*τ*_*income*,*s*_ = *β*_1_ + *β*_4_ and *τ*_*soccap*,*s*_ = *β*_2_ + *β*_5_). For instance, if social capital ranges on a scale from 0 to 4, we add the interaction coefficients of each level *s* to the main effects.
3. we compute the moderation effect (*φ*) of each level of social capital *s* as follows:
  $$\varphi_{js}=100-\frac{\tau_{js}\cdot 100}{\beta_{m}}$$
  where *j* stands for own income and social comparisons, respectively, and *m* indicates the two main effects. We compute the standard errors of the moderation effects using the error propagation model. For details, please refer to the online S1 Appendix.

## 3 Data

We draw data from four, freely available and widely used datasets. Available measures of social comparisons include reference income, income rank, and self-reported social class. These measures reflect individuals’ position compared to others in society. Reference income and income ranks are income-based measures of social comparisons. Self-reported social class provides a subjective appraisal of one’s social status. In particular, reference income, measured as the average income of the reference group, is a frequently used measure of social comparisons because it is a benchmark against which individuals evaluate their income levels. Income ranks follow the same reasoning, as respondents state their income using income brackets based on the national income distribution. Self-reported social class is a measure of comparisons because it involves individuals comparing their own social status to that of others in their social and economic context, and it refers to their perception of their own social position in society.

The use of both income-based and self-reported measures of social comparisons allows us to account for two approaches to the way individuals select comparison targets [35]. The first approach assumes that objective characteristics such as proximity (e.g. area of residence) or similarity (gender, age, etc.) determine comparison targets. The second approach underlines the role of subjective perceptions and preferences in the selection of comparison targets from a range of possible alternatives.

We exploit the European Union Survey on Income and Living Conditions (EU-SILC), the European Social Survey (ESS), the integrated European Values Study—World Values Study (EVS-WVS), and the German Socio-Economic Panel (SOEP). Besides being freely available and known to most social scientists, these datasets allow us to test the robustness of our findings to a variety of samples, wordings and measures of subjective well-being, social capital and social comparisons. The first three datasets provide internationally comparable data, which allow us to test our hypothesis on a rich set of countries. SOEP provides panel data from Germany, and allows us to account for individual fixed effects.

### 3.1 European Union Statistics on Income and Living Conditions

The European Union Statistics on Income and Living Conditions (EU-SILC) is a general population survey mandated by the European Commission to collect timely and internationally comparable data on income, social exclusion and living conditions in European Union member states.

The EU-SILC is a yearly survey with a rolling sample and rotating modules. The rolling sample ensures that part of the sample can be followed longitudinally for a maximum of four years. The rotating modules permit to collect data on specific topics. In particular, information about subjective well-being and social capital were first administered in 2013 and subsequently in 2018. However, the latter wave of data has not been made available for research. Therefore, we use cross-sectional micro-data from the 2013 EU-SILC for the purposes of present analysis.

The EU-SILC (2013) sample includes approximately 318,000 observations coming from 29 European countries (Table A4 in the S1 Appendix provides detailed descriptive statistics). The data provide three variables related to subjective well-being, namely life satisfaction, frequency of feeling downhearted or depressed, and job satisfaction. We are aware that job satisfaction is a measure of satisfaction in a specific life domain, rather than an encompassing evaluation of life as a whole. Nonetheless, we decided to include it in the analysis because it is an important aspect of people’s life, and one that can be easily affected by social comparisons, especially on the workplace.

Life satisfaction is observed through answers to the question: “Overall, how satisfied are you with your life these days? Please answer on a scale of 0 to 10, where 0 means ‘Not at all satisfied’ and 10 means ‘Completely satisfied’.” The second measure of (lack of) subjective well-being is based on answers to the question: “How much of the time over the past four weeks have you been downhearted and depressed? Please answer on a scale from 1 to 5, where 1 means ‘All of the time’ and 5 means ‘None of the time’.” We reverted the scores so that higher values indicate higher ill-being. Job satisfaction follows the same wording of the question about life satisfaction, but asks explicitly about present work. Also in this case the answers range on a scale of 0 to 10, where 0 means ‘Not at all satisfied’ and 10 means ‘Completely satisfied’.” As job satisfaction pertains to people in employment, our analysis is restricted to a sub-sample of workers made of about 152,000 individuals.

Our main explanatory variable is social capital. The EU-SILC provides two measures of social capital, trust in others and frequency of meeting with friends, which we combine in a single index. The trust question asks: “Would you say that most people can be trusted? Please answer on a scale from 0 to 10, where 0 means that in general ‘You do not trust any other person’ and 10 that you feel ‘Most people can be trusted’.” We construct a dummy variable equal to one for answers larger than five, the median value, zero otherwise. The frequency of meeting with friends is based on the answers to the question: “Do you meet up with friends/family for a drink/meal (at home or outside) at least once a month? (Yes/No)”. We build a dummy variable equal to one if an individual meets his friends or family at least once per month, zero otherwise. The social capital index simply adds up the two dummies. Hence, the index is a categorical variable taking values from zero to two, where higher values stand for more social capital.

Income is the monthly disposable equivalised income adjusted to purchasing power parities by country. The equivalised disposable income is the total income of a household, after tax and other deductions, that is available for spending or saving, divided by the number of equivalent adults. Household members are made equivalent by weighting each of them using the so-called modified OECD equivalence scale. The scale applies a weight of 1.0 to the first adult; 0.5 to the second and each subsequent person aged 14 and over; 0.3 to each child aged under 14. To correct for purchasing power parities we use price level indices for the actual individual consumption (EU28 = 100) from Eurostat.

We proxy social comparisons with reference income. This variable is computed as the average income of the reference group. We assume that respondents compare their incomes with those of other people of the same sex and age group living in the same region. This definition provides a total of 990 reference groups. The average number of individuals in a reference group is about 312.

To account for individual heterogeneity, we use a standard set of control variables including respondent’s age, gender, marital status, education level, occupation, home ownership, being chronically sick or disabled, i.e. an objective measure of health [36, 37] and the country of residence. This set of socio-demographic characteristics is common to all the datasets available for present analysis. The only exception is the control for health which, in case of the Integrated World Values Survey—European Values Study (WVS-EVS), is self-reported subjective health. As this variable is likely to be endogenous to subjective well-being, we do not control for health status in the analysis of WVS-EVS data. The detailed list of control variables by dataset is provided in the note to Table A1 in the S1 Appendix.

### 3.2 European Social Survey

The European Social Survey (ESS) is a bi-annual survey administered in various European countries since 2002. Each wave of the ESS provides internationally comparable, and nationally representative data on adult population. It provides a rich set of information about people’s lives, feelings, values and preferences. Specifically, the ESS provides data on income, life satisfaction and happiness, various measures of social capital, along with other individual level data.

We use the 9th round of the European Social Survey which was administered in 2018. This is the latest available wave before the pandemic (2020). In each wave the ESS randomly interviews about 2000 individuals per country. In 2018 the sample included about 38,000 individuals from 29 European countries. Table A12 in the S1 Appendix provides detailed descriptive statistics.

The ESS provides two measures of subjective well-being, life satisfaction and happiness. Both variables record respondents’ answers using a 0 to 10 scale where higher scores indicate higher well-being. Life satisfaction is observed through answers to the question: “All things considered, how satisfied are you with your life as a whole nowadays? Please answer using this card, where 0 means ‘extremely dissatisfied’ and 10 means ‘extremely satisfied’.” The wording of the happiness question is: “Taking all things together, how happy would you say you are? 0 Extremely unhappy, 10 Extremely happy”.

As for the measure of social capital, we use similar proxies to those used in the analysis of EU-SILC data. The answers to the question “How often do you meet socially with friends, relatives or work colleagues?” are recoded in a dummy variable set to one if a respondent meets socially at least once per week. The ESS provides three questions that provide an overall evaluation of how much respondents trust others. The wordings are: “Generally speaking, would you say that most people can be trusted, or that you can’t be too careful in dealing with people?”; “Do you think that most people would try to take advantage of you if they got the chance, or would they try to be fair?”; and “Would you say that most of the time people try to be helpful or that they are mostly looking out for themselves?”. Answers range on a scale from zero to ten, in which higher scores indicate higher levels of perceived trustworthiness, fairness, and helpfulness. After factor analysis, we compute a synthetic index of social trust by averaging the answers to each question. Subsequently, we create a dummy variable (labelled “social trust”) set equal to one if the synthetic index ranges between 6 and 10, zero otherwise. Finally, we create the index of social capital as the sum of the dummies about frequency of meeting friends, and social trust. The index takes values from the set *s* = 0, 1, 2 where higher values indicate more social capital.

The ESS questionnaire invites the respondent to choose the interval corresponding to his or her household’s total income. There are ten intervals which are country specific and delimited by income deciles. In other words, each income interval is relative to the national income distribution. Thus, our proxy of social comparisons is the income rank, i.e. the individual’s position in the national income distribution. Income rank is a categorical variable and, for the sake of simplicity when used with interactions, we recoded it in three levels: income rank 1–3 (for the bottom three deciles), income rank 4–7 (for the middle four deciles), and income rank 8–10 (for the top three deciles).

As for income, we impute the disposable household monthly income by attributing to each respondent the average household income of the income bracket to which he/she declares to belong to (the original variable is the same used for income rank). In the case of non-Euro countries we convert the new variable to euros. Subsequently, we adjust for purchasing power parity (PPP) using the conversion factor provided by Eurostat (EU28 = 100).

### 3.3 Integrated World Values Survey—European Value Study

The World Values Survey (WVS) and the European Value Study (EVS) are two widely explored datasets made of repeated cross-sectional surveys that started in 1981. Although separate, the two surveys can be integrated, as they are largely harmonized. The integrated WVS-EVS covers roughly every country in the World, and provides a nearly unique source of comparative information about people’s feelings, believes, values, and attitudes.

At present the WVS and the EVS comprise respectively 7 and 6 waves, covering the period 1981—2021. In particular, the 3rd, 5th and 6th waves of the World Values Survey—European Values Study integrated dataset provide the sole source of free data that we are aware of with information about self-reported social class. We use this information to directly observe respondent’s relative placement in a society. In the course of the interviews, respondents are asked the following question: “People sometimes describe themselves as belonging to the working class, the middle class, or the upper or lower class. Would you describe yourself as belonging to the: 1. Upper class; 2. Upper middle class; 3. Lower middle class; 4. Working class; 5. Lower class; 6. No answer”. We record the last category to missing.

The three selected waves provide information about 417,000 respondents from 68 countries world-wide. For the purposes of present analysis, we focus on a subset of 20 developed countries, for a total of nearly 50,000 respondents. Table A27 in the S1 Appendix provides detailed descriptive statistics, and Table A30 in S1 Appendix lists the countries included in the analysis.

The integrated WVS-EVS provides two proxies of subjective well-being, namely life satisfaction, and happiness. The wording for the former is: “All things considered, how satisfied are you with your life as a whole these days?” Possible answers range on a 1 to 10 scale in which the lowest value corresponds to “dissatisfied” and the highest to “satisfied”. Happiness is observed via answers to the following question: “All considered you would say that you are: 1. very happy; 2. pretty happy; 3. not too happy; 4. not at all happy?” This variable has been recoded so that the category “very happy” corresponds to the highest value in the scale, and the category “not at all happy” corresponds to the lowest one.

Our measure of social capital follows the same specification adopted in the analysis of EU-SILC and ESS data. In particular, the WVS-EVS integrated dataset contains information about trust in others based on the following question: “Generally speaking, would you say that most people can be trusted, or that you can’t be too careful in dealing with people?”, with answers coded as 1 (“most people can be trusted”), and 0 (“you can’t be too careful”). Additionally, we consider respondents’ participation in various groups and associations. During interviews, people are asked whether they are members or not of a list of groups or associations. We created a dummy variable taking values of 1 if the respondent declares to participate in at least one Putnam’s group or association, 0 otherwise. Putnam and colleagues [24] identify in associations a source of general trust and of social ties leading to governmental and economic efficiency. Among Putnam’s groups we include: social welfare service for elderly, church organizations, sport clubs, art and literature clubs, fraternal groups and youth associations, human and animal rights. Finally, we create the index of social capital as the sum of the two dummies. The index takes values from the set *s* = 0, 1, 2, where higher values indicate more social capital.

Respondents of the WVS and EVS are asked to specify to which income bracket they belong to. Differently from the ESS, however, the income brackets do not necessarily reflect income deciles of the national income distribution. Therefore, this variable cannot be regarded as income ranking. Moreover, there is considerable variety in the way this question is administered across countries and time. Therefore, we kept its original categorical scale without applying any transformation.

### 3.4 German Socio-Economic Panel

The SOEP is a panel dataset administered yearly in Germany by the DIW. It was first administered in 1984 in West Germany and, as of June 1990, its sample widened to include households and individuals from East Germany. The main focus of the SOEP is to monitor demographic, economic, social and political aspects of life in Germany. Although the data span a long time period, our analysis is limited by the years when the survey recorded information about Germans’ social capital. Thus, our data cover the period from 1990 to 2011. The sample consists of 9 waves for a total of about 36,600 individuals interviewed at least two times, giving more than 129,900 observations. Table A26 in the S1 Appendix provides detailed descriptive statistics.

The wording of the question about life satisfaction is fairly similar to those used in previous surveys. Specifically, the questions reads: “Please answer on a scale from 0 to 10, where 0 means ‘completely dissatisfied’ and 10 means ‘completely satisfied’: How satisfied are you with your life, all things considered?”.

Our main explanatory variable, the index of social capital, is defined as the sum of four dummy variables: “Attending social gatherings”, “Helping friends”, “Performing volunteering work”, and “Participating in local politics”. Each dummy variable is set equal to one if the respondent carries out a given activity at least once per month, zero otherwise. Thus, the social capital index ranges from zero (for individuals not performing any of the activities), to four (for people who perform all four activities).

Income is defined as monthly equivalised disposable income, and it is adjusted by the price level in a given year (transformed in logarithm). We use reference income to proxy for social comparisons. Reference income is computed as the average income (in logarithmic form) of the reference group. We assume that respondents compare their incomes with those of other people of the same sex, age group and living in the same geographical area (West or East Germany) in the same year. In total we have 210 reference groups (ten reference groups per year for the three waves before unification (which do not include East Germany), and twenty reference groups per year for the nine waves after unification). The average number of respondents per reference group is 755.

## 4 Results

We present our results in terms of moderation effects, i.e. the percentage by which the original coefficients of income and social comparisons decrease when they are interacted with social capital. Essentially, moderation effects indicate by how much the association between income and well-being change, for different levels of social capital. These effects are based on the coefficients estimated from Eq 1, which are available in Table A2 in the S1 Appendix for brevity.

Table 1 reports the moderation effects for all the measures of subjective well-being (life satisfaction, happiness, feeling depressed, job satisfaction) available in the EU-SILC (first panel), European Social Survey (second panel) and the WVS-EVS (third panel, last two rows). Table 2 reports the moderation effects computed on the SOEP dataset.

**Table 1:** Moderation effects of social capital in the relationships between own income and social comparisons with subjective well-being. Results from cross-sectional data.

	Life Satisfaction		Happiness		Depressed		Job Satisfaction	
	(SC index = 1)	(SC index = 2)	(SC index = 1)	(SC index = 2)	(SC index = 1)	(SC index = 2)	(SC index = 1)	(SC index = 2)
European Union Statistics on Income and Living Conditions								
Own income	-16% *** (0,0334)	-47% *** (0,0221)			-37% *** (0,0461)	-54% *** (0,0334)	-39% *** (0,04542)	-58% *** (0,0325)
Reference income	-28% *** (0,1077)	-94% *** (0,1770)			-56% *** (0,1025)	-96% *** (0,1492)	-54% *** (0,0926)	-69% *** (0,1274)
European Social Survey								
Own income	-29% *** (0,0766)	-52% *** (0,0700)	-53% *** (0,0694)	-66% *** (0,0715)				
Income rank 1–3	-6% (0,4771)	-119% *** (0,3458)	89% (1,52)	-76% (0,5954)				
Income rank 8–10%	-43% (0,3202)	-77% *** (0,2430)	-105% *** (0,327)	-128% *** (0,3176)				
Integrated World Values Survey—European Values Study								
Own income	-20% ** (0,084)	-55% *** (0,0669)	-21% (0,1978)	-50% *** (0,2208)				
Social Class	-33% *** (0,058)	-52% *** (0,054)	-27% *** (0,0741)	-38% *** (0,0681)				

Note: Moderation effects indicate by how much each level of the social capital index reduces the income coefficients of the subjective well-being regression.

Method: OLS regression with robust standard errors of the three proxies of subjective well-being. Errors (in parentheses) are estimated using the error propagation method.

**Table 2:** Moderation effects of social capital in the relationships of own income and social comparisons with subjective well-being. Results from longitudinal data.

	Life Satisfaction			
	(SC index = 1)	(SC index = 2)	(SC index = 3)	(SC index = 4)
Own income	-17% ** (0,073)	-27% *** (0,0802)	-44% *** (0,0771)	-52% *** (0,1129)
Reference income	-35% ** (0,1327)	-61% *** (0,1743)	-67% *** (0,1724)	-79% *** (0,2642)

Note: Moderation effects indicate by how much each level of the social capital index reduces the income coefficients of the subjective well-being regression. Method: OLS regression with robust standard errors and individual fixed effects. Errors (in parentheses) are estimated using the error propagation method.

The results consistently exhibit large moderation effects. For each level of social capital—presented across columns—the moderation effect is smaller for own income than for social comparisons, as measured by reference income, income rank and social class.

For example, taking results from the EU-SILC (column 1 of Table A2 in the S1 Appendix), the coefficient of “Social capital index = 2 * income” is -0.241, while the coefficient of “income” is 0.511, meaning that for those who have high social capital (SCindex = 2) the income effect is −0.241 + 0.511 = 0.27. This indicates that the original income coefficient has decreased by 0.241, which in percentage terms is equal to 0.241/0.511 = 47.16% (as seen in column 2 of Table 1, on the first row referring to income). Similarly for the ESS, results indicate that for people with high social capital (SC = 2), the negative income rank (1–3) effect −0.161 becomes −0.161 + 0.191 = 0.03, which in percentage terms is 0.191/0.161 = 119%. In this case the negative effect of comparing to people higher up in the income distribution is completely offset for people who have high levels of social capital. Similarly, the positive coefficient of social comparison for the richest people (compared to people in the middle income group) is around 77% lower (0.120/0.156 = 0.769) when they have high social capital. In sum, the correlation between own income and subjective well-being reduces by nearly 50 percent for people with the highest levels of social capital. In most cases the correlation between social comparisons and subjective well-being is nearly entirely off-set for people with high social capital.

These findings indicate that the subjective well-being of socially isolated people (those with social capital index = 0) depends nearly twice as much on own income than the subjective well-being of socially active individuals (i.e., those with maximum level of the social capital index). Vice-versa, social comparisons show a strong association with the subjective well-being of individuals with little social capital.

The results from German panel data provide a consistent picture. The first row of Table 2 indicates that the association between own income and subjective well-being decreases when individuals’ social capital increases. The moderation effects range from around -17% for people with low social capital to almost -53% for people with the highest level of social capital. The moderation effects of social capital in the relation between social comparisons, as measured by reference income, and subjective well-being are consistently larger than those for own income. Moderation effects range from around -35% for people with low social capital, to almost -80% for people with high social capital.

In the remaining of this section, we briefly describe the other results from Eq 1, which are available in Table A2 in the S1 Appendix. Coefficients of the income variable indicate that higher income correlates with greater subjective well-being in all datasets. For instance, the income coefficients in the EU-SILC are *b* = 0.511*** for life satisfaction, *b* = 0.675*** for job satisfaction and *b* = −0.156*** for depressive feelings and in the ESS they amount to *b* = 0.529*** for life satisfaction and *b* = 0.491*** for happiness. A one standard deviation (SD) increase in log income is associated to an increase between 0.2 and 0.4 SD of life satisfaction for the cross section results, and 0.125 SD in the case of SOEP.

Coefficients also indicate that social comparisons have, in general, a negative association with subjective well-being: the coefficients on the reference income variable in the EU-SILC are negative and statistically significant for life and job satisfaction, and positive for depressive feelings, as well as for life satisfaction in the SOEP dataset. One standard deviation increase in log reference income in the EU-SILC and SOEP is associated to a reduction in life satisfaction SDs of 0.135 and 0.05, respectively.

In the ESS, social comparisons are proxied by three categories of income rank, 1–3, 4–7 and 8–10. We use income rank 4–7 as reference category, so that people who belong to the lowest (1–3) and the highest (8–10) income rank both compare to those in the middle of the income distribution. Hence, the negative coefficient on the lowest income rank (income rank 1–3 = −0.161) indicates that people at the lower end of the income distribution compare to others who are richer than them, which lowers their subjective well-being. By contrast, the coefficient for people at the upper end of the income distribution (income rank 8–10 = 0.156), implies that richer people compare to others who are lower in the income distribution and this has a positive effect on their subjective well-being. Subjective well-being is lower for the respondents to the EVS-WVS who declared to belong to the lowest social class.

As for the remaining variables, we find the typical U-shaped relation between age and subjective well-being; married or cohabiting people report—on average—higher subjective well-being than single ones; having an illness negatively correlates with well-being, as do being divorced or separated and being unemployed. Being a student, retired and owning a house instead correlate positively with subjective well-being. The main effects of social capital are positively associated to subjective well-being.

The collinearity between social capital and income variables could raise some concerns about the reliability of our results. A Variance Inflation Factor test for multicollinearity shows that multicollinearity is not a concern (VIF is consistently below 5, as seen in Table A1 in the S1 Appendix). This result is reassuring also because of the self-reported nature of independent and dependent variables. The correlation between our variables of interest could be inflated because of unobservable features such as personality traits, or similar response scales, that rise the probability of common method bias when respondents’ answers are systematically correlated, thus inflating the results [38, 39]. Additionally, correlation tables between own income, measures of social comparisons and social capital are available for each dataset in the S1 Appendix (please, refer to Tables A4, A16, A26, A34 in S1 Appendix).

## 5 Accounting for endogeneity

The results from Eq 1 can be spurious and affected by endogeneity issues, as the association between social capital and subjective well-being may be driven by omitted variables or reverse causality. We try to address this issue using a Two-Stages Least Squares (2SLS) instrumental variable approach. Specifically, we instrument the main effect of social capital, and its interaction terms with own income and social comparisons using the method of heteroskedasticity generated instruments proposed by Lewbel [40]. Finding a proper instrument for social capital is difficult as most of the factors affecting people’s social life will likely affect their subjective well-being as well. The Lewbel approach allows to identify a causal model without imposing the exclusion restriction which is typically required in a standard 2SLS, while instead exploiting the internal structure of the data—that is, the heteroskedasticity of the first step equation—to construct the instruments [40]. This approach has been used numerous times (as documented in Lewbel [40]), in applied economics settings such as health, education and happiness economics [41–46]. One downside of this approach is that the generated instruments do not have an economic meaning. This limitation is mentioned in the original paper by Lewbel [40].

We provide a detailed description of the method and the formal model in the S1 Appendix. For more details, please, refer to Lewbel and Baum and Lewbel’s contributions [40, 47]. In brief, we generate the instruments **Z** as follows: 1) we run a regression of each endogenous variable on the full set of controls from Eq 1 and store the residuals $\hat{\mu }$; 2) these residuals are multiplied by the demeaned vector of the controls (or a subset of them) to construct the instruments $Z_{j}=\left(X_{j}-\bar{X_{j}}\right)\cdot \hat{\mu }$. If the chosen *Xs* are exogenous, by construction, the covariance between the residuals $\hat{\mu }$ and the demeaned controls is zero, but with heteroskedasticity the instruments **Z** will take meaningful values.

The method relies on two conditions in addition to those that are standard in instrumental variables (IV) approach. The first is that there exists heteroskedasticity in the first stage equation, that is *Cov*(**Z**, *μ*^2^) ≠ 0, which we test with a a Breusch-Pagan test. The non-constant functional form that social capital takes over different age groups may be a likely cause of heteroskedasticity in the first step. For instance, people in different age groups may attach different importance to social capital, or have more/less time to dedicate to social interactions (i.e. there is a non-constant variance in the effects age exerts on social capital). The second assumption is that the covariance between the product of the error terms in the structural and reduced form equations and the vector of controls, *Cov*(**Z**, *μϵ*), is zero. Although the second condition is untestable, we use the typical IV diagnostics to assess whether the instruments are relevant (first stage F-statistics) and valid (Hansen-J overidentification test). Hence, in order for the instruments to be correctly identified they need to hold information on the variation of the endogenous variable—that is, they come from the heteroskedasticity in the model –, and should be constructed on controls that are exogenous to the dependent variable in the structural equation. Among the set of controls we choose age and age squared, as these are exogenously determined with respect to life satisfaction. In this choice of controls on which to construct the instruments, our approach is similar to Elsas [46]. The instruments are created with the STATA user written command ivreg2h from Baum and Schaffer [48].

To limit the number of instruments necessary for our 2SLS estimations, we use the index of social capital as a continuous variable. We remind the reader that we have three endogenous variables in our specification: social capital and its two interactions with social comparisons and own income. By using the index of social capital as a continuous variable we can limit the number of instruments necessary for identification to six (or eight in case of the ESS).

We use a 2SLS model even if subjective well-being is not a continuous variable as the coefficients estimated with a linear model are comparable to the marginal effects produced by non linear instrumental variable models [49]. We expect the coefficient on social capital to be biased upwards. Indeed, if we assume a bias given by the omission of unobserved personality traits, the direction with which they affect social capital and life satisfaction is likely the same. For instance, a more extrovert person may be more likely to have an active social life, but also be happier. Likewise, a neurotic person will probably tend to have less social capital and lower levels of subjective wellbeing.

We perform this analysis on all four datasets, but we run the regression uniquely on life satisfaction for parsimony. Additionally, life satisfaction is the most commonly used proxy of subjective well-being in the literature, it is generally thought to be more reliable than other proxies, and lastly because it is the only measure that is common and available for each of our four datasets. Our results, however, hold also when using the other proxies of subjective well-being. Results are available in the Table A38 in S1 Appendix.

Table 3 shows the coefficients of the regression using Lewbel’s method. Columns 1 to 4 report the results of the analysis on EU-SILC, ESS, WVS-EVS and SOEP data, respectively. We report only the results for the main variables of interest for brevity. The complete set of results is available in the Tables A7, A17, A25 and A37 in S1 Appendix.

**Table 3:** Results accounting for endogeneity using two-stage least square regressions with generated instruments.

	Life Satisfaction			
	EU-SILC (1)	ESS (1)	WVS-EVS (1)	SOEP (1)
Own income	0.710*** (0.0788)	0.920*** (0.170)	0.103*** (0.0302)	1.614*** (0.416)
Reference Income	-0.484*** (0.113)			-1.437*** (0.255)
Income Rank 1–3		0.104 (0.316)		
Income Rank 8–10		-0.379 (0.305)		
Social Class			-0.713*** (0.221)	
Social capital * Own income	-0.270*** (0.0607)	-0.555*** (0.176)	-0.0255 (0.0280)	-0.812*** (0.275)
Social Capital * Reference Income	0.269*** (0.0832)			0.656*** (0.150)
Social Capital * Income rank 1–3		-0.237 (0.311)		
Social Capital * Income Rank 8–10		0.460 (0.289)		
Social Capital * Social Class			0.427** (0.212)	
Social Capital	0.821*** (0.277)	4.542*** (1.318)	-0.849 (0.698)	1.099 (1.745)
Number of Observations	317978	38597	48849	119701
Adjusted R	0.3129	0.2434	0.1293	-0.0084
Overidentification test: Hansen Statistics	1.834	5.501	9.295	1.562
HJ P-value	0.6075	0.2396	0.1577	0.6680
First step F test: Social capital	143.07	0.68	15.44	36.98
First step F test: Social capital*own income	36.07	0.63	126.51	16.29
First step F test: Social capital*reference income	53.87		14.48	235.63
First step F test: Social capital*income rank 1–3		61.6		
First step F test: Social capital*income rank 8–10		112.37		

Note:

* *p* < 0.05,

** *p* < 0.01,

*** *p* < 0.001,

s.e. in parentheses. Instrumented variables are the “social capital”, “social capital * own income” and “social capital * reference income”. The method is 2SLS with robust standard errors, where the employed instruments have been generated using the Lewbel method.

The social capital variable is treated as continuous to limit the number of instruments necessary for identification.

Own income is log of income for the EU-SILC and SOEP, log of household income for ESS and household income for WVS-EVS.

Reference income is log of reference income for EU-SILC and GSOEP, income rank for ESS and self reported social class for WVS-EVS.

Controls included in each of the estimated equations are the same of those included in the main OLS results.

Fixed effects are included in the SOEP.

The coefficients from the 2SLS applied to the EU-SILC and SOEP datasets support our previous findings: own income attracts a positive and significant coefficient (*b* = 0.71 *** and *b* = 1.61 ***, respectively); social comparisons, measured as reference income (row 2) attracts negative and significant coefficients. The interaction between own income and social capital suggests that the association between own income and subjective well-being is smaller for people with higher social capital; importantly, the coefficient of the interaction between social capital and reference income is always positive and significant suggesting that social capital moderates the effects of social comparisons for subjective well-being.

The moderation effects are similar to those found using the OLS method shown in the previous section, for which social capital significantly moderates both own income and reference income in the EU-SILC and SOEP datasets. Moderation effects after 2SLS suggest that the coefficients of both own income and reference income nearly half (between 38% and 56%) for people with high levels of social capital. Table A37 in the S1 Appendix provides the full set of moderation effects.

The first stage weak identification F statistics for the relevance of the instruments are presented at the bottom of the table. These are computed for the individual endogenous regressors. A rule of thumb indicates that an acceptable F statistics would be greater than 10 [50]. Our results for the tests are in almost all cases well above the recommended threshold. This suggests that our endogenous variables are properly instrumented with non-weak instruments, except for the results estimated on the ESS dataset. Moreover, the Hansen J-statistic and the associated p-values, presented right under the Adjusted *R*^2^, indicate that the null hypothesis of the instruments being valid is not rejected.

## 6 Well-being inequality and social capital

Individual level results indicate that own income and social comparisons are less strongly associated to subjective well-being in individuals with high social capital than in others. Specifically, back of the envelop calculations indicate that the weighted subjective well-being gap between rich and poor people is smaller (−0.145) for people with high social capital than for others (‘weighted’ refers to the fact that the difference has been divided by the national average life satisfaction to account for the mechanic association between average and dispersion [51, 52]. The unweighted difference amounts to about 1 life satisfaction point. For more details, please, see the S1 Appendix, in section 1.1).

If this is the case, we should expect that, at aggregate level, income differences should be less related to subjective well-being differences in countries with high social capital. In this section, we check whether empirical evidence supports this expectation. We focus on life satisfaction as our main measure of subjective well-being.

There are two key variables in this case. First, the share of people with high social capital (which we define as the share of respondents with a social capital index equal to two). In our samples, the share of people with high social capital is 46% in EU-SILC, 28% in the ESS and WVS-EVS, and 3% (for the highest level, SC = 4) in the SOEP, but around 30% for people with SC = 2. The second key variable is the weighted life satisfaction gap between rich and poor people. The gap is the difference in the average life satisfaction between the first and fifth income quintile by country, divided by the national average in life satisfaction. Both variables are computed by country. In case of EU-SILC data, we repeat the analysis also at regional level. Descriptive statistics of the country characteristics may be found in the S1 Appendix.

**Fig 1:**
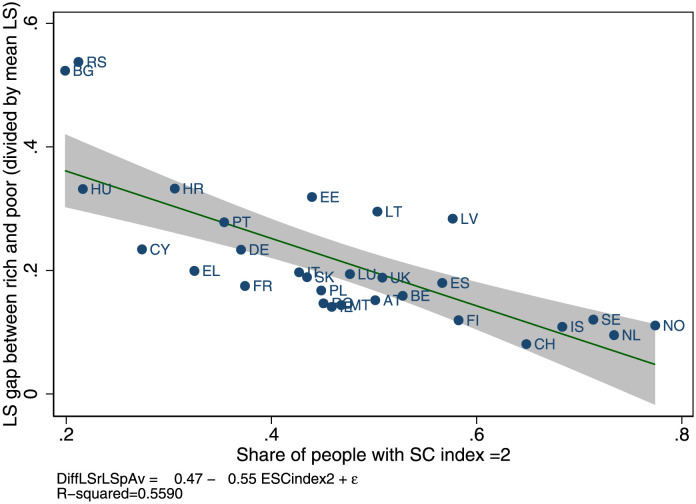
Weighted life satisfaction gap between rich and poor people across European countries. Note: 29 European countries; data EU-SILC 2013. Social capital is measured as the share of respondents with a social capital index = 2. The social capital index has a maximum score of 2 if a person trusts others and meets friends at least once per month. Life satisfaction ranges on a 0 to 10 scale, where largest scores stand for higher life satisfaction. Country-level scores are divided by average life satisfaction to account for the mechanic relation between average and dispersion. Aggregated data are computed from individual data using sample weights.

**Fig 2:**
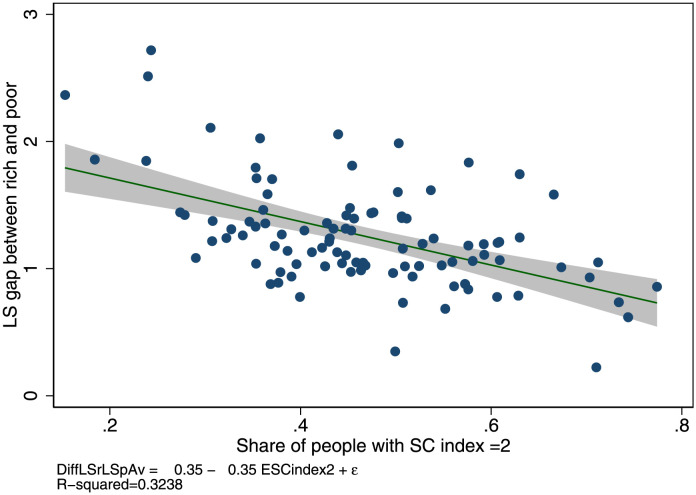
Weighted life satisfaction gap between rich and poor people across European regions. Note: 99 European regions; data EU-SILC 2013. Social capital is measured as the share of respondents with a social index equal to 2. The social capital index has a maximum score of 2 if a person trusts others and meets friends at least once per month. Life satisfaction ranges on a 0 to 10 scale, where largest scores stand for higher life satisfaction. Regional scores are divided by average life satisfaction to account for the mechanic relation between average and dispersion. Aggregated data are computed from individual data using sample weights.

Figs 1 and 2 provide descriptive results. Across 29 European countries and 99 regions the weighted life satisfaction gap between the rich and poor people is smaller in countries with high social capital than in others. For instance, in Serbia and Bulgaria, where social capital is very low (around 20% of people have the highest level, as seen in the upper left corner in Fig 1), the weighted life satisfaction gap between rich and poor people is 0.5, whereas in countries with high social capital—such as Switzerland or the Netherlands (see the lower right corner)—it is around 0.08, i.e. a difference of about 0.42.

Such differences could be affected by income inequality, which is greater in Serbia and Bulgaria than in Switzerland or the Netherlands. As own income is a well-established correlate of subjective well-being—as confirmed by our micro analysis—it is straightforward to expect that its distribution affects the distribution of subjective well-being. Moreover, a more skewed income distribution tends to exacerbate social comparisons, amplifying well-being differences between income groups [53, 54]. To account for these possible confounding effects, we turn to regression analysis.

Our measure of income inequality is the Gini index of equivalized disposable income, as provided by Eurostat [55]. For the analysis at regional level, we compute the regional Gini index as the sample weighted income by region, using the EU-SILC 2013 micro data. GDP per capita for European countries is sourced from Eurostat [56], and is expressed in thousands of current euro corrected for purchasing power parity. We do not correct for inflation because the data refer to 2013 for the EU-SILC and to 2018 for the ESS. The analyses on the integrated WVS-EVS, and ESS data use figures from the World Development Indicators of the World Bank [57]. In this case GDP per capita is expressed in thousands of constant US dollars for the WVS-EVS, and in current international dollars corrected for purchasing power parity for the ESS.

Formally, we test the following equation using OLS with robust standard errors:

$$\begin{array}{c} \Delta LS_{c}=\alpha +\beta_{1}\cdot log\left(GDPpercapita_{c}\right)+\beta_{2}\cdot Gini_{c}+\beta_{3}\cdot SC_{c}+\varepsilon_{c} \end{array}$$ (2)

where the unit of analysis are countries (*c*), Δ*LS* is the life satisfaction gap between people belonging to the first and to the fifth income quintile in a given country divided by the average life satisfaction in the country, and *SC* is the share of people with high social capital (SC index = 2). We expect the social capital variable to attract a negative coefficient, indicating that a greater share of individuals with high social capital is associated to a smaller life satisfaction gap. We control for the Gini index of the income distribution to account for the likely influence of income differences on well-being differences. Moreover, we control for countries’ GDP, as more prosperous countries are expected to exhibit lower life satisfaction inequality [58]. All variables have been standardised for comparability.

Holding constant the Gini index of the income distribution and GDP per capita, countries and regions where social capital is higher exhibit a smaller life satisfaction gap between rich and poor people than elsewhere (see Table 4, which reports the results of Eq 2).

**Table 4:** Weighted life satisfaction gap and social capital controlling for the Gini index of income and GDP per-capita.

	Weighted life Satisfaction gap between rich and poor			
	(1)	(2)	(3)	(4)
	EU-SILC Region	EU-SILC country	WVS-EVS	ESS
Share of people with social capital index = 2	-0.521 *** (0.108)	-0.486 ** (-3.14)	-0.131 (-1.83)	-0.248 (-1.22)
Gini index	0.245* (0.111)	0.296* (2.34)	-0.0821 (-0.32)	0.255 (1.71)
GDP per capita (log)	-0.0416 (0.0683)	-0.145 (-1.41)	0.00566 (0.03)	-0.356 (-1.37)
*N*	99	29	60	29

Note: *t* statistics in parentheses.

* *p* < 0.05,

** *p* < 0.01,

*** *p* < 0.001.

The unit of analysis are countries (except in Column 1, in which the unit of analysis are regions).

The dependent variable is the difference in average life satisfaction between the first and fifth income quantile in a given country divided by average life satisfaction to account for the mechanic relation between average and dispersion.

All coefficients are standardised for comparability. The regression using WVS-EVS data includes time fixed effects to account for the fact that some countries are observed multiple times. The sample available for the regression is N = 60 because some of the countries have been observed more than once.

Aggregated figures are captured from individual data using sample weights.

Method: OLS regression with robust standard errors.

For instance, in the country-level analysis of EU-SILC data, one standard deviation increase in the share of people with high social capital is associated with half a standard deviation decrease in the weighted life satisfaction gap between rich and poor people. This means that the weighted life satisfaction gap between rich and poor people would decrease by 0.054 (0.486*0.112, where 0.112 is the standard deviation of the weighted life satisfaction gap between rich and poor people), when the share of people with high social capital increases by one standard deviation. The same results indicate that if we increased social capital from the minimum to the maximum level observed in our sample, the predicted weighted life satisfaction gap between rich and poor people would decrease by 0.2 points, which is very close to the 0.15 points we estimated based on the individual-level regressions (please, see S1 Appendix, section 1.1 for more details). The predicted unweighted difference is 0.96 life satisfaction points, which is very close to the 1 point difference we computed using micro-level data.

Moreover, we find a positive correlation between income inequality and weighted life satisfaction gap, though significant only in the analysis carried out on the EU-SILC data (the estimated coefficient for Gini index is 0.245 in column 1 and 0.296 in column 2). We also find that the effect of GDP per capita is not statistically significant (see the last row of Table 4). These results are robust to alternative measures of inequality, such as the 90/10 and 50/10 income inequality ratios, that is the ratio between the average income of the 90th (50th) percentile with respect to the one of the 10th percentile (results are available in Tables A10-A13, A21, A22 and A31 in S1 Appendix): all other things being equal, the higher the share of individuals with high social capital, the lower the weighted life satisfaction gap between the rich and the poor (see the coefficients in the first row of Table 4). The same result holds if life satisfaction gap is not weighted by the average life satisfaction. These cross-country/region results reflect the micro-level findings presented previously. The more income is associated to life satisfaction, the more income disparities translate into life satisfaction disparities between income groups.

## 7 Discussion

Our finding has three implications. First, results support the view that social comparisons and social capital are strictly intertwined. This view was initially expressed by Veblen, and found later support in the quantitative findings of positive psychologists and Barcena-Martin and colleagues. This is also consistent with previous studies documenting that declining social capital coexisted with increasing social comparisons over decades in countries experiencing economic growth and decreasing subjective well-being, such as China and the US [23, 59, 60]. We add to this literature by widening the generality of previous conclusions. Moreover, we extend our analysis to own income, showing that social capital moderates its relationship with subjective well-being. To the best of our knowledge, this is an original contribution.

The second implication concerns the origin of social comparisons. Some studies suggest that social comparisons are rooted in human evolution and in the biology of the brain [61–63]. Our result indicates that this may not be the whole story: the social context affects the importance of social comparisons for subjective well-being. Previous studies also documented that social comparisons can negatively affect people’s physical [64–67] and mental health [66, 68–70], as well as their economic decisions [71–74]. Our finding suggests that promoting social capital could mitigate the negative consequences of social comparisons. This could be beneficial in particular for those who more likely lose from social comparisons.

The third implication of our finding is that social capital changes the extent to which income inequality affects subjective well-being inequality. The more closely income and subjective well-being are connected, the more a given income inequality should produce subjective well-being inequality. Our findings indicate that the closeness of such connection is negatively related to social capital.

In our individual-level regressions, the moderation effect of social capital on the relationship between own income and subjective well-being is never complete, suggesting that the income distribution shapes the well-being distribution even in the presence of high levels of social capital, although to a lesser extent.

This is reflected in the cross-country evidence we provide in section 6: holding constant the income distribution and gross domestic product, the life satisfaction gap between rich and poor people—a measure of well-being inequality—is larger in countries with low social capital. This suggests that policies to promote social capital may reduce the inequality of subjective well-being associated to income inequality. We emphasize, however, that this does not mean that promoting social capital can replace redistribution policies: a well-being difference between rich and poor people persists even in countries with high social capital. Moreover, income inequality facilitates social comparisons regardless of social capital [53, 54, 75], and high inequality can hamper social capital [29, 76–78]. For these reasons highly unequal societies tend to exhibit low social capital and strong social comparisons. Thus, redistributive policies have a crucial role, independently from social capital, because limited inequality is a prerequisite for a society capable of expanding the well-being of its members.

Finally, we note that social comparisons are the main factor driving the disappointing impact of economic growth on subjective well-being [79, 80]. As the economy grows, social comparisons erode part of its benefits for subjective well-being. Our findings suggest that promoting social capital is key to weaken social comparisons, besides reducing income inequality. Alleviating the well-being consequences of social comparisons by promoting social capital would allow economic growth to unfold its potential to increase subjective well-being. This view is consistent with previous evidence showing that economic growth correlates with increasing subjective well-being over time when social trust does not decline and income inequality does not increase [81].

If social capital is important, how can it be promoted? Domains such as urban planning, education, and advertising devoted considerable attention to policies for social capital. For instance, according to New Urbanism, an urban design movement, planning cities and neighborhoods with high residential density, walkability, pedestrian areas, parks, car restrictions and public transport can contrast the effects of car-oriented urban development. Re-organizing common spaces and transport is critical to relieve the urban car-dependency, and promoting social capital [82]. Long commutes take a high relational toll: people who spend more than 45 minutes commuting are less happy than others, and they are 40 percent more likely to divorce [83]. Studies comparing traditional high-density neighborhoods and conventional low-density suburbs find greater social interaction and sense of community in traditional neighborhoods, and availability of pedestrian areas increased the likelihood of social interactions [84, 85]. Other studies focused directly on the degree of walkability [86] and demonstrated that more walkable neighborhoods enhance social interactions and a greater sense of community [29, 85, 87–91]. Gilderbloom and coauthors have shown that walkability has a positive impact not only on neighborhoods’ social fabric, but also on real estate prices, foreclosures and even crime rates [92]. Walkable neighborhoods translate into more “eyes on the street”, which contributes to safer living spaces.

Evidence from education studies shows that children’s education heavily affects the development of the social skills that are critical for the development of social capital later in life. Current teaching practices, mostly based on vertical teaching, contribute to make education a distressing and competitive experience for most students [93]. Participatory teaching practices are an effective alternative. Participatory teaching is based on students’ group work on common projects, in student-centered classrooms and has been shown to foster students’ social capital in the forms of cooperation with other students and teachers, membership in associations, trust in institutions, and participation in civil society [94]. Predictably, more cooperation-oriented schooling practices shape more cooperative individuals. The foundations of participatory teaching were laid by Montessori education—a century-old schooling method [95]. Lillard and Else-Quest found that Montessori education fosters social and academic skills more than traditional education [96].

Lastly, advertising negatively affects social capital and increases social comparisons, especially for children and teenagers. Studies have documented a relationship between exposure to advertising and materialism in children [97–101]. By triggering feelings of exclusion in those who do not buy the advertised products [97], advertising promotes social comparisons. Similar to adults, children’s materialism is bad for their social capital: it is associated with family conflict, less generosity and more anti-social behaviour [20, 100–103]. Increasing awareness of the damage caused by commercial pressure has lead various Western countries to regulate advertising. Norway and Greece banned television advertisements targeting kids, New Zealand prohibits advertising of junk food and Austria and Belgium have banned ads targeting kids before, during or after children’s TV programs. Authorities for the regulation of advertising are at the forefront in regulating children’s media in countries such as Australia, Canada, and the UK [104, 105]. Advertising fosters social comparisons among adults as well; thus, regulating advertising would benefit adults too.

## 8 Conclusion

In this paper we investigate whether the relationship between income and subjective well-being depends on individual’s social capital. We analyse the role of social capital in relation to income as a mean to satisfy individual’s needs (own income), and as a source of social comparisons. We apply regression analysis with interaction effects to four international and freely available datasets. Our results suggest that own income and social comparisons are less strongly associated to subjective well-being for people with high social capital compared to others. The moderation effects, i.e. the amount by which income and social comparisons’ coefficients reduce for people with high social capital, are above 50% in case of own income (all datasets); above 70% for reference income (EU-SILC and SOEP), and income rank (ESS); and between 37.7% and 51.8% in case of social class (WVS-EVS) for happiness and life satisfaction, respectively. These results are robust to a test of endogeneity using Lewbel’s method of generated instruments. Lastly, we found supportive evidence for a country-level implication of our findings: if income were all that mattered to subjective well-being, then richer people should be happier than poorer ones. However, as the association between own income and social comparisons with subjective well-being is weaker for people with high social capital, then countries in which social capital is high should exhibit lower well-being gaps between rich and poor people. We find that the life satisfaction gap between rich and poor people is smaller in countries with high social capital compared to others.

The empirical approach used in this study has some limitations. First, statistical identification of a causal relation is challenging. As it is often the case, exogenous sources of variation are scarce and it is difficult to identify the direction of causality. However, the individual-based evidence is encouraging: the results obtained using the method of generated instruments lend some support to a causal interpretation of our findings. A second limitation relates to the use of large samples, which comes at the expense of not having a rich battery of questions to measure social capital.

On the up-side, present findings seem robust to a variety of countries, measures, wordings of the variables of interest, measures of social comparisons, and of social capital. Our findings also provide encouraging news on the role of social comparisons for subjective well-being. Promoting social capital may be an effective strategy to minimize the competitive consumption that typically characterises social comparisons. As social comparisons are the main reason for the disappointing impact of economic growth on subjective well-being, promoting social capital could allow growth to fully display its potential to increase subjective well-being. Previous studies documented that social capital contributes to happiness, health, social cohesion, resilience and economic prosperity. According to our findings, the list of the beneficial effects of social capital should include also the moderation of social comparisons.

## Supporting information

S1 Appendix Online appendix. Contains robustness checks and additional figures.
(PDF)

S1 File Files to replicate the Tables and Figures in this article. (ZIP)
