# Supplementary material for: The moderation effect of social capital in the relationship between own income, social comparisons and subjective well-being: Evidence from four international datasets
Source: PLoS One. 2023 Dec 7;18(12):e0288455. doi: 10.1371/journal.pone.0288455 (PMC10703203; doi:10.1371/journal.pone.0288455)
Supplement: S1 Appendix — Contains robustness checks and additional figures. (PDF) [file pone.0288455.s001.pdf]

S1 Appendix. Online Appendix

Table A1: VIF Test

	EU-SILC	ESS	WVS-EVS	SOEP
Social capital = 1	2.46	1.67	1.72	3.25
Social capital = 2	2.76	1.87	2	3.33
Social capital = 3				1.98
Social capital = 4				1.29
individual income	12.33	6.05	1.56	341
reference income	303.53		1.43	402.04
income rank 1-3		2.82		
income rank 8-10		1.73		

Note: The high collinearity on the income and reference income should not cause any concern, as it is a mechanical consequence of the construction of the reference income variable.

Table A2: The role of social capital in the relationship between absolute income and social comparisons with subjective wellbeing. Regression results from four datasets.

		EU-SILC			ESS		WVS-EVS		SOEP
		(1)	(2)	(3)	(4)	(5)	(6)	(7)	(8)
		Life satisfaction	Depressed	Job Sat.	Life satisfaction	Happiness	Life satisfaction	Happiness	Life satisfaction
Variables in common	Absolute income	0.511***	-0.156***	0.675***	0.529***	0.491***	0.108***	0.0179***	0.474***
		(0,019)	(0,009)	(0,042)	(0,034)	(0,046)	(0,009)	(0,003)	(0,041)
	Reference income	-0.158***	0.105***	-0.397***					-0.698***
		(0,035)	(0,018)	(0,064)					(0,145)
	(Social capital index = 1) * absolute income	-0.0806***	0.0582***	-0.268***	-0.153***	-0.261***	-0.0210**	-0.00383	-0.0824**
		(0,02)	(0,010)	(0,0454)	(0,051)	(0,047)	(0,011)	(0,004)	(0,041)
	(Social capital index = 2) * absolute income	-0.241***	0.0911***	-0.392***	-0.274***	-0.327***	-0.0583***	-0.00968***	-0.127***
		(0,02)	(0,009)	(0,044)	(0,054)	(-0,050)	(0,012)	(0,004)	(0,043)
	(Social capital index = 3) * absolute income								-0.207***
									(0,049)
	(Social capital index = 4) * absolute income								-0.248***
									(-0,063)
EU-SILC	(Social capital index = 1) * reference income	0.0442**	-0.0589***	0.213***					
		(0,02)	(0,009)	(0,046)					
	(Social capital index = 2) * reference income	0.148***	-0.101***	0.276***					
		(0,02)	(0,0096)	(0,055)					
European Social Survey	Income rank 1-3				-0.161**	-0.0804			
					(0,069)	(0,064)			
	Income rank 8-10				0.156**	0.105*			
					(0,062)	(0,056)			
	(Social capital index = 1) * Income rank 1-3				0.0102	-0.0713			
					(0,081)	(0,073)			
	(Social capital index = 1) * Income rank 8-10				-0.0665	-0.110*			
					(0,070)	(0,062)			
	(Social capital index = 2) * Income rank 1-3				0.191**	0.0615			
					(0,083)	(0,075)			
	(Social capital index = 2) * Income rank 8-10				-0.120*	-0.134**			
					(0,070)	(0,062)			
WVS-EVS	Social class (subjective)						-0.381***	-0.103***	
							(0,024)	(0,008)	
	(Social capital index = 1) * Social class (subjective)						0.125***	0.0277***	
							(0,028)	(0,009)	
	(Social capital index = 2) * Social class (subjective)						0.197***	0.0393***	
							(0,029)	(0,009)	
Socio-Economic Panel	(Social capital index = 1) * reference income								0.246**
									(0,114)
	(Social capital index = 2) * reference income								0.429***
									(0,121)
	(Social capital index = 3) * reference income								0.466***
									(0,143)
	(Social capital index = 4) * reference income								0.550***
									(0,200)
	Social capital index = 1	1.168***	-0.304***	0.960***	1.808***	2.629***	-0.0636	-0.00946	-0.995
		(0.0317)	(0.0151)	(0.0582)	(0.388)	(0.363)	(0.123)	(0.0404)	(0.835)
	Social capital index = 2	2.154***	-0.447***	1.842***	3.227***	3.520***	0.235*	0.0834**	-1.960**
		(0.0342)	(0.0163)	(0.0599)	(0.417)	(0.381)	(0.126)	(0.0424)	(0.874)
	Social capital index = 3								-1.619
									(1.041)
	Social capital index = 4								-1.933
									(1.424)
	Controls	Yes	Yes	Yes	Yes	Yes	Yes	Yes	Yes
	Constant	4.399***	2.612***	5.082***	3.573***	3.801***	8.071***	3.854***	9.434***
		(0.224)	(0.121)	(0.367)	(0.402)	(0.377)	(0.179)	(0.059)	(1.05)
	Number of observations	317978	317978	152095	38597	38597	48849	49973	129901
	Adjusted R^2	0.315	0.171	0.120	0.253	0.221	0.147	0.121	0.0585

Note: all regressions are estimated with OLS with robust standard errors. t statistics in parentheses. * $p < 0.05$, ** $p < 0.01$, *** $p < 0.001$

Omitted categories: "Social capital = 0", "Social capital = 0 * log of absolute income", "Social capital = 0 * log of reference income" for EU-SILC. "Social capital = 0", "Social capital = 0 * log of household income", "Social capital = 0 * income rank 4-7" for ESS. "Social capital = 0", "Social capital = 0 * household income", "Social capital = 0 * social class" for WVS-EVS. "Social capital = 0", "Social capital = 0 * log of absolute income", "Social capital = 0 * log of reference income" for SOEP.

Controls: Gender, age group, marital status, educational level, labour market status, house owner, country dummies, person has a permanent disability (EU-SILC); Gender, age, age squared, living with partner, have children, years of education, disabled, labour market status (ESS); Gender, age, age squared, education, marital status, number of children, labour market status, country and year dummies (WVS-EVS); Gender, age, age squared, marital status, years of education, labour market status, house owner, disabled, living in East Germany, regional dummies, year dummies (SOEP).

The estimated model for the SOEP is a panel OLS regression with Fixed Effects, while the other datasets are cross-sections.

A VIF test for multicollinearity of social capital shows values lower than 5 for each dataset.

1 EU-SILC

Table A3: OLS with robust standard errors using EU-SILC (2013) data: detailed results.

	(1) Life Sat.		(2) Depression feeling		(3) Job Sat.	
Social capital index = 1	1.168***	(0.0317)	-0.304***	(0.0151)	0.960***	(0.0582)
Social capital index = 2	2.154***	(0.0342)	-0.447***	(0.0163)	1.842***	(0.0599)
Log of individual income	0.511***	(0.0195)	-0.156***	(0.00883)	0.675***	(0.0422)
Social capital index = 1 * Log of individual income	-0.0806***	(0.0218)	0.0582***	(0.00992)	-0.268***	(0.0454)
Social capital index = 2 * Log of individual income	-0.241***	(0.0208)	0.0911***	(0.00959)	-0.392***	(0.0440)
Log of reference income	-0.158***	(0.0352)	0.105***	(0.0181)	-0.397***	(0.0636)
Social capital index = 1 * Log of reference income	0.0442**	(0.0220)	-0.0589***	(0.00996)	0.213***	(0.0457)
Social capital index = 2 * Log of reference income	0.148***	(0.0211)	-0.101***	(0.00970)	0.276***	(0.0443)
Female	0.105***	(0.00674)	0.122***	(0.00350)	0.0487***	(0.0102)
26-35	-0.311***	(0.0170)	0.105***	(0.00870)	-0.0342	(0.0254)
36-45	-0.521***	(0.0176)	0.156***	(0.00894)	-0.0169	(0.0255)
46-55	-0.704***	(0.0183)	0.188***	(0.00931)	-0.0471*	(0.0268)
Above 55	-0.565***	(0.0188)	0.111***	(0.00957)	-0.00232	(0.0279)
Married	0.405***	(0.00960)	-0.0842***	(0.00501)	0.194***	(0.0131)
Widowed	-0.00154	(0.0156)	0.0541***	(0.00799)	0.160***	(0.0387)
Divorced or separated	-0.0894***	(0.0146)	0.0608***	(0.00747)	0.168***	(0.0207)
has disability	-0.578***	(0.00751)	0.302***	(0.00390)	-0.305***	(0.0131)
Secondary education	0.131***	(0.0122)	-0.0734***	(0.00622)	0.0448	(0.0307)
Tertiary education	0.294***	(0.0137)	-0.113***	(0.00698)	0.172***	(0.0316)
Unemployed	-0.861***	(0.0153)	0.314***	(0.00752)		
Student	0.308***	(0.0178)	0.000981	(0.00915)		
Retired	0.0373***	(0.0112)	-0.0237***	(0.00577)		
Not working	-0.231***	(0.0123)	0.119***	(0.00630)		
House owner	0.143***	(0.00796)	-0.0443***	(0.00409)	-0.00360	(0.0127)
Constant	4.399***	(0.224)	2.612***	(0.120)	5.082***	(0.367)
Number of observations	317978		317978		152095	
Adjusted R^2	0.315		0.171		0.120	

Notes: Dependent variable: Life satisfaction (0-10). Depressed = frequency of feeling depressed or downhearted (1-5). Job Sat. = Job satisfaction (1-10).
Omitted categories: "Social capital index = 0", "Social capital index = 0 * log of individual income", "Social capital index = 0 * log of reference income".
* $p < 0.05$, ** $p < 0.01$, *** $p < 0.001$. Standard errors in parenthesis.

Table A4: Correlations table

	Getting together with friends	individual income	Trust in others	reference income	SCindex
Getting together with friends	1				
individual income	0.2463*	1			
Trust in others	0.1398*	0.1156*	1		
reference income	0.1890*	0.9496*	0.0883*	1	
SCindex	0.7243*	0.2342*	0.7840*	0.1793*	1

Table A5: Robustness check using the single dummies for social capital rather than the index (EU-SILC, 2013

	Life Satisfaction		Depression		Job satisfaction	
	(1)	(2)	(3)	(4)	(5)	(6)
	Getting together with friends	Trust in others	Getting together with friends	Trust in others	Getting together with friends	Trust in others
SC	1.224***	1.193***	-0.240***	-0.265***	0.999***	1.074***
	(0.0268)	(0.0258)	(0.0127)	(0.0117)	(0.0457)	(0.0424)
Log of individual income	0.493***	0.616***	-0.146***	-0.165***	0.623***	0.569***
	(0.0142)	(0.0116)	(0.00633)	(0.00506)	(0.0286)	(0.0191)
SC * Log of individual income	-0.121***	-0.277***	0.0590***	0.0786***	-0.268***	-0.222***
	(0.0157)	(0.0134)	(0.00705)	(0.00603)	(0.0304)	(0.0224)
Log of reference income	-0.109***	-0.172***	0.0793***	0.0704***	-0.303***	-0.256***
	(0.0333)	(0.0320)	(0.0173)	(0.0167)	(0.0562)	(0.0518)
SC * Log of reference income	0.0533***	0.213***	-0.0616***	-0.0775***	0.190***	0.150***
	(0.0160)	(0.0139)	(0.00720)	(0.00623)	(0.0309)	(0.0230)
Constant	4.623***	4.476***	2.568***	2.672***	5.117***	5.265***
	(0.228)	(0.227)	(0.121)	(0.120)	(0.369)	(0.366)
Controls (socio-demographic, country)	Yes	Yes	Yes	Yes	Yes	Yes
Observations	317978	317978	317978	317978	152095	152095
Adjusted R^2	0.285	0.294	0.156	0.160	0.102	0.111

Note: OLS with robust standard errors. Dependent variable: Life satisfaction (0-10). Depressed = frequency of feeling depressed or downhearted (1-5). Job Sat. = Job satisfaction (1-10).
Controls: sex, age group, marital status, education level, labour market status, house owner, long standing illness or disability, country dummies. * $p < 0.05$, ** $p < 0.01$, *** $p < 0.001$. Standard Errors in parenthesis.

Table A6: Descriptive statistics (EU-SILC, 2013): micro-data

	count	mean	sd	min	max
Life satisfaction	317978	6.939401	2.133051	0	10
Depressed (1-5)	317978	2.039849	1.004411	1	5
Job satisfaction (0-10)	152095	7.245071	2.053106	0	10
Individual income	317978	1140.575	1260.038	0	110438.8
Log of individual income	317978	6.159619	1.909749	0	11.61223
Reference income	317978	1135.363	766.3204	1.982637	3120.429
Log of reference income	317978	6.308553	1.828514	1.092808	8.046046
Getting together with friends	317978	.734142	.4417897	0	1
Trust in others	317978	.5772695	.4939941	0	1
Social capital index (0-2)	317978	1.311411	.706143	0	2
Social capital index = 0	317978	.142101	.3491543	0	1
Social capital index = 1	317978	.4043865	.4907737	0	1
Social capital index = 2	317978	.4535125	.497835	0	1
Female	317978	.5506702	.4974267	0	1
Under 26	317978	.0961828	.2948422	0	1
26-35	317978	.1259112	.3317498	0	1
36-45	317978	.1682443	.3740837	0	1
46-55	317978	.1891389	.3916195	0	1
Above 55	317978	.4205228	.4936437	0	1
Single	317978	.2459069	.4306243	0	1
Married	317978	.5671807	.495467	0	1
Widowed	317978	.0973338	.2964122	0	1
Divorced or separated	317978	.0895785	.2855774	0	1
has disability	317978	.352603	.4777812	0	1
Primary education or no education	317978	.1260024	.3318526	0	1
Secondary education	317978	.6277101	.4834158	0	1
Tertiary education	317978	.2462875	.4308486	0	1
Working	317978	.4758914	.4994192	0	1
Unemployed	317978	.0754926	.264185	0	1
Student	317978	.0545352	.2270711	0	1
Retired	317978	.2779029	.447966	0	1
Not working	317978	.1161778	.3204386	0	1
House owner	317978	.5581235	.4966109	0	1

Table A7: Lewbel EU-SILC

	(1)
	LifeSat
Social Capital Index	0.821***
	(0.277)
Social capital * individual income	-0.270***
	(0.0607)
Social capital * reference income	0.269***
	(0.0832)
Individual Income	0.710***
	(0.0788)
Reference Income	-0.484***
	(0.113)
Female	0.103***
	(0.00678)
Age	-0.0662***
	(0.00162)
Age Squared	0.0606***
	(0.00161)
Married	0.417***
	(0.00995)
Widowed	-0.0677***
	(0.0167)
Divorced or Separated	-0.0611***
	(0.0149)
Secondary Education	0.147***
	(0.0149)
Tertiary Education	0.306***
	(0.0192)
Unemployed	-0.832***
	(0.0194)
Student	0.200***
	(0.0177)
Retired	-0.0313**
	(0.0126)
Not working	-0.224***
	(0.0140)
House Owner	0.131***
	(0.00914)
has disability or longstanding illness	-0.573***
	(0.0106)
Constant	6.427***
	(0.425)
County Fixed effects	Yes
N	317978
Adjusted R^2	0.3129
Hansen Statistic	1.834
p-value	0.6075
First step F test: Social Capital	143.07
First step F test: Social Capital * individual income	36.07
First step F test: Social Capital * reference income	58.87
Kleibergen-Paap Wald F test	87.825

Standard errors in parentheses
* $p < 0.10$, ** $p < 0.05$, *** $p < 0.01$

Table A8: Descriptive statistics: EU-SILC (2013), country level data.

	count	mean	sd	min	max
LS gap between 1st and 5th income quintile / average LS	29	.2150211	.1126427	.0808457	.5372779
Share of people with SC index =2	29	.4673399	.1544134	.1987608	.7739409
Gini index	29	30.20345	4.033652	22.7	38
GDP per capita	29	27.17931	12.49118	10.1	70.5

Table A9: Descriptive statistics: EU-SILC (2013), regional level data.

	count	mean	sd	min	max
LS gap between 1st and 5th income quintile / average LS	99	.1838056	.0790098	.031316	.5570245
Share of people with SC index =2	99	.4644989	.1273024	.1529882	.7739409
Gini index	99	.2954481	.0362704	.2300008	.4260471
GDP per capita	99	24.79495	8.545059	9.3	70.5

Table A10: Robustness check of country level analysis on EU-SILC (2013) data using the 50/10 ratio as a measure of income inequality.

	Difference in life satisfaction between and poor / average LS		
	(1)	(2)	(3)
Share of people with SC = 2	-0.486**	-0.582***	-0.552***
	(0.142)	(0.132)	(0.142)
Gini	0.296*		
	(0.127)		
GDP per capita (log)	-0.145	-0.127	-0.0666
	(0.103)	(0.100)	(0.0909)
50/10 share		0.333*	
		(0.150)	
50/10 cut-off			0.373
			(0.202)
N	29	28	28

Note: Standard errors in parentheses.
* $p < 0.05$, ** $p < 0.01$, *** $p < 0.001$
Note: The unit of analysis are countries. All coefficients are standardised for comparability.
Data for Gini, 50/10 ratio and GDP are from Eurostat. Share refers to share of national equivalised income and cut-off refers to the top cut-off point. All variables are standardised for comparability.

Table A11: Robustness check of regional level analysis on EU-SILC (2013) data using the 50/10 ratio as a measure of income inequality.

	Difference in life satisfaction between rich and poor / average LS		
	(1)	(2)	(3)
Share of people with SC = 2	-0.521***	-0.622***	-0.628***
	(0.108)	(0.132)	(0.130)
Gini	0.245*		
	(0.111)		
GDP per capita (log)	-0.0416	-0.0176	0.0355
	(0.0683)	(0.0688)	(0.0642)
50/10 share		0.195*	
		(0.0919)	
50/10 cut-off			0.258*
			(0.114)
N	99	97	97

Note: Standard errors in parentheses.
* $p < 0.05$, ** $p < 0.01$, *** $p < 0.001$
Note: The unit of analysis are countries. All coefficients are standardised for comparability.
Data for Gini, 50/10 ratio and GDP are from Eurostat. Share refers to share of national equivalised income and cut-off refers to the top cut-off point. All variables are standardised for comparability.

Table A12: Robustness check of country level analysis on EU-SILC (2013) data using the 90/10 ratio as a measure of income inequality.

	Difference in life satisfaction between rich and poor / average LS		
	(1)	(2)	(3)
Share of people with SC = 2	-0.486**	-0.588***	-0.516**
	(0.141)	(0.130)	(0.152)
Gini	0.296*		
	(0.127)		
GDP per capita (log)	-0.145	-0.0467	-0.0642
	(0.101)	(0.0820)	(0.0825)
90/10 share		0.400*	
		(0.146)	
90/10 cut-off			0.396*
			(0.183)
N	29	28	28

Standard errors in parentheses
* $p < 0.05$, ** $p < 0.01$, *** $p < 0.001$
Note: The unit of analysis are regions. All coefficients are standardised for comparability.
Data for Gini, 90/10 ratio and GDP are from Eurostat. Share refers to share of national equivalised income and cut-off refers to the top cut-off point.
All variables are standardised for comparability.

Table A13: Robustness check of regional level analysis on EU-SILC (2013) data using the 90/10 ratio as a measure of income inequality.

	(1)	(2)	(3)
Share of people with SC = 2	-0.521***	-0.641***	-0.613***
	(0.108)	(0.131)	(0.124)
Gini	0.245*		
	(0.111)		
GDP per capita (log)	-0.0416	0.0173	0.0464
	(0.0683)	(0.0636)	(0.0571)
90/10 share		0.242*	
		(0.0955)	
90/10 cut-off			0.275*
			(0.106)
N	99	97	97

Standard errors in parentheses

* $p < 0.05$, ** $p < 0.01$, *** $p < 0.001$

Note: The unit of analysis are regions. All coefficients are standardised for comparability. Data for Gini, 90/10 ratio and GDP are from Eurostat. Share refers to share of national equivalised income and cut-off refers to the top cut-off point.
All variables are standardised for comparability.

1.1 Size effects

Well-being differences between rich and poor people form micro-results and macro results: the aim is to relate micro- to macro- results by comparing the size of estimated effects of the subjective well-being differences between rich and poor people (corrected for by dividing this difference by the average life satisfaction). We are interested in understanding what is the difference in life sfatisfaction between income groups (top and bottom) when people have high social capital, and the same difference when people have no social capital. The expectation is that this difference would be higher in people who have no social capital. We are intterested in the following reduced computation:

$$((\bar{L}S_{rich} - \bar{L}S_{poor})|SC = 2) - ((\bar{L}S_{rich} - \bar{L}S_{poor})|SC = 0) = \beta_{SC=2*income} + \beta_{SC=2*SocComp} \quad (1)$$

In terms of making these results comparable to between micro and macro estimations we do the following:

- We compute the average levels income of the top and bottom income quintiles and multiply them by the coefficient of the interaction effect of social capital (=2) and income, to get the the effect at the mean: $-0.24 * 7.87 = -1.89$ and $-0.24 * 2.97 = -0.72$
- similarly, we compute the average reference income of people in the top and bottom income quintiles, and multiply it by the interaction coefficient of the social capital (=2) and reference income ($0.148 * 7.50 = 1.11$ and $0.148 * 3.36 = 0.49$)
- So the life satisfaction gap between top and bottom income quintiles with high social capital, minus the life satisfaction gap of rich and poor people having no social capital, corrected of the average levels of life satisfaction to obtain relative differences is equal to $((-1.89 + 1.11) + (-0.7 + 0.49))/averageLifeSatisfaction$, which is almost -0.145. This suggests that the life satisfaction gap between top and bottom income quintiles would be reduced by 0.145 for people with high levels of social capital, compared to well-being differences of people with no social capital.

- At the macro level, we run the unstandardized regression of life satisfaction gap between the top and bottom income quintiles divided by average life satisfaction on the share of people with high social capital, Gini index and GDP, and multiply the coefficient on the share of high social capital people (-0.354) with the difference in shares of high social capital people in countries at the maximum level and the minimum level in the sample, namely: $(0.774 - 0.198) * -0.354 = -0.0204$ (as seen in table A8, second row). This suggests that increasing the share of people in a country will reduce the life satisfaction gap between the top and bottom income quintiles.

2 European Social Survey

Table A14: LS with robust standard errors using the ESS 2018

	(1)		(2)	
	Life satisfaction (0-10)		Happiness (0-10)	
Social capital index = 1	1.808***	(0.388)	2.629***	(0.363)
Social capital index = 2	3.227***	(0.417)	3.520***	(0.381)
Income rank 1-3	-0.161**	(0.0696)	-0.0804	(0.0637)
Income rank 8-10	0.156**	(0.0623)	0.105*	(0.0555)
Social capital index = 1 * Income rank 1-3	0.0102	(0.0802)	-0.0713	(0.0727)
Social capital index = 1 * Income rank 8-10	-0.0665	(0.0702)	-0.110*	(0.0625)
Social capital index = 2 * Income rank 1-3	0.191**	(0.0824)	0.0615	(0.0747)
Social capital index = 2 * Income rank 8-10	-0.120*	(0.0693)	-0.134**	(0.0618)
Household income	0.529***	(0.0484)	0.491***	(0.0456)
Social capital index = 1 * Household income	-0.153***	(0.0505)	-0.261***	(0.0473)
Social capital index = 2 * Household income	-0.274***	(0.0537)	-0.327***	(0.0492)
Sex (1=male)	-0.0886***	(0.0198)	-0.140***	(0.0178)
Age	-0.0548***	(0.00399)	-0.0450***	(0.00358)
Age squared (divided by 100)	0.0521***	(0.00398)	0.0397***	(0.00359)
Years of education	0.0179***	(0.00265)	0.0127***	(0.00238)
Unemployed	-0.698***	(0.0577)	-0.368***	(0.0523)
Student	0.218***	(0.0493)	0.205***	(0.0444)
Retired	0.00551	(0.0380)	-0.0851**	(0.0340)
Not working	-0.0260	(0.0440)	-0.0266	(0.0381)
Living with partner	0.438***	(0.0251)	0.591***	(0.0225)
Has children	0.0856***	(0.0281)	0.129***	(0.0251)
Permanently sick or disabled	-0.980***	(0.0746)	-0.716***	(0.0656)
Constant	3.573***	(0.402)	3.801***	(0.377)
Number of observations	38597		38597	
Adjusted ^{2}	0.253		0.221	

* $p < 0.05$, ** $p < 0.01$, *** $p < 0.001$. Standard errors in parentheses.
Note: Dependent variables: Life Satisfaction (1-10), Happiness (1-10).
Omitted categories: "Income rank 4-7", "Social Capital index = 0 * Log of household income" and "Social Capital index = 0 * income rank 4-7". Included controls are also country dummies

Table A15: Robustness check using the single dummies of social capital rather than the index (ESS 2018).

	(1) Life satisfaction (0-10)	(2) Life satisfaction (0-10)	(3) Happiness (0-10)	(4) Happiness (0-10)
Meeting socially		1.921***		2.655***
		(0.311)		(0.289)
Social trust	1.797***		1.520***	
	(0.301)		(0.276)	
Household income	0.459***	0.516***	0.347***	0.464***
	(0.0366)	(0.0405)	(0.0341)	(0.0380)
Income rank 1-3	-0.151***	-0.166***	-0.142***	-0.0769
	(0.0481)	(0.0554)	(0.0436)	(0.0506)
Income rank 8-10	0.131***	0.140***	0.0611*	0.0419
	(0.0396)	(0.0440)	(0.0357)	(0.0394)
Social trust * Household income	-0.146***		-0.125***	
	(0.0385)		(0.0353)	
Meeting socially * Household income		-0.180***		-0.276***
		(0.0399)		(0.0371)
Social trust * Income rank 1-3	0.117**		0.110**	
	(0.0594)		(0.0534)	
Social trust * Income rank 8-10	-0.0828*		-0.100**	
	(0.0468)		(0.0420)	
Meeting socially * Income rank 1-3		0.0776		-0.0587
		(0.0635)		(0.0574)
Meeting socially * Income rank 8-10		-0.0818		-0.0469
		(0.0504)		(0.0450)
Controls (socio-demographic, country)	Yes	Yes	Yes	Yes
Number of observations	38597	38597	38597	38597
Adjusted R^2	0.239	0.233	0.202	0.203

Standard errors in parentheses * $p < 0.05$, ** $p < 0.01$, *** $p < 0.001$
Note: OLS with robust standard errors
Controls: sex, age, age squared, living with partner, having children, years of education, permanently sick or disabled, labour market status, country dummies.

Table A16: ESS: Correlations Table

	Meeting socially	Social trust	Income rank	Household income	Social capital index
Meeting socially	1				
Social trust	0.1032*	1			
Income rank	0.0661*	0.1282*	1		
Household income	0.1250*	0.2284*	0.7220*	1	
Social capital index	0.7393*	0.7460*	0.1309*	0.2382*	1

Table A17: Lewbel ESS

	(1)
	LS
Social capital index	4.542***
	(1.318)
Social Capital * Household income	-0.555***
	(0.176)
Social Capital * Income rank 1-3	-0.237
	(0.311)
Social Capital * Income rank 8-10	0.460
	(0.289)
Household income	0.920***
	(0.170)
Income rank 1-3	0.104
	(0.316)
Income rank 8-10	-0.379
	(0.305)
Female	0.0946***
	(0.0204)
Age	-0.0571***
	(0.00418)
Age Squared (divided by 100)	0.0540***
	(0.00413)
Child	0.0837***
	(0.0286)
Unemployed	-0.707***
	(0.0581)
Student	0.218***
	(0.0499)
Retired	0.0110
	(0.0383)
Not working	-0.0239
	(0.0443)
Years of education	0.0207***
	(0.00320)
Permanently sick or disabled	-1.012***
	(0.0773)
Partner	0.422***
	(0.0271)
Constant	1.011
	(1.228)
Country dummies	Yes
Number of observations	38597
Adjusted R^2	0.2434
Hansen Statistic	5.501
p-value	0.2394
First step F test: Social Capital	0.68
First step F test: Social Capital * individual income	0.63
First step F test: Social Capital * income rank 1-3	61.6
First step F test: Social Capital * income rank 8-10	112.37
Kleibergen-Paap Wald F test	7.016
Endogeneity test p-value	0.1679

Standard errors in parentheses

* $p < 0.10$, ** $p < 0.05$, *** $p < 0.01$

Additional controls are country dummies.

Table A18: Descriptive statistics (ESS 2018).

	count	mean	sd	min	max
Life satisfaction (0-10)	38597	7.14698	2.177084	0	10
Happiness (0-10)	38597	7.456875	1.912693	0	10
Household income (EUR, EU28=100)	38597	7.527611	.8426338	4.839056	9.718941
Income rank 1-3	38597	.318859	.4660403	0	1
Income rank 4-7	38597	.4230122	.4940437	0	1
Income rank 8-10	38597	.2581289	.4376109	0	1
Social trust	38597	.4467446	.4971622	0	1
Meeting socially	38597	.6038293	.489107	0	1
Social capital index (0-2)	38597	1.050574	.7342374	0	2
Social capital index = 0	38597	.2455372	.4304108	0	1
Social capital index = 1	38597	.4583517	.4982689	0	1
Social capital index = 2	38597	.2961111	.4565465	0	1
Female	38597	.5303262	.4990859	0	1
Age	38597	51.90593	18.06593	15	90
Age squared (divided by 100)	38597	30.20594	18.83742	2.25	81
Years of education	38597	13.09444	4.191266	0	60
Permanently sick or disabled	38597	.0337073	.180477	0	1
Working	38597	.5205068	.4995858	0	1
Unemployed	38597	.0477498	.2132392	0	1
Student	38597	.0529834	.2240032	0	1
Retired	38597	.2871208	.4524243	0	1
Not working	38597	.0916392	.2885198	0	1
Living with partner	38597	.5968858	.4905297	0	1

Table A19: Detailed results of the country level analysis using ESS (2018) data.

	Life Satisfaction gap between rich and poor/ average LS			
	(1)	(2)	(3)	(4)
Share of people with SC index =2	-0.633***			-0.248
	(0.139)			(0.202)
Gini index (std.)		0.524*		0.255
		(0.208)		(0.149)
GDP per capita (log)			-0.670**	-0.356
			(0.196)	(0.260)
Number of observations	29	29	29	29
Adjusted R^2	0.400	0.274	0.449	0.529

Standard errors in parentheses

* $p < 0.10$, ** $p < 0.05$, *** $p < 0.01$

Note: The unit of analysis are countries.

Data for Gini are from Eurostat, data for GDP are from the World Bank.

All variables are standardised for comparability.

Table A20: Descriptive statistics: ESS (2018), country level data.

	count	mean	sd	min	max
LS gap between rich and poor / average LS	29	.1636586	.085511	.0618447	.4143289
Social capital index (0-2)	29	.2854172	.1656544	.0863853	.6047104
Gini index (Eurostat)	29	29.50345	4.512798	20.9	39.6
GDP per capita, PPP	29	10.6571	.3377325	9.978005	11.34935

Figure A1: Across European countries, the life satisfaction gap between rich and poor people negatively correlates with social capital (ESS, 2018).

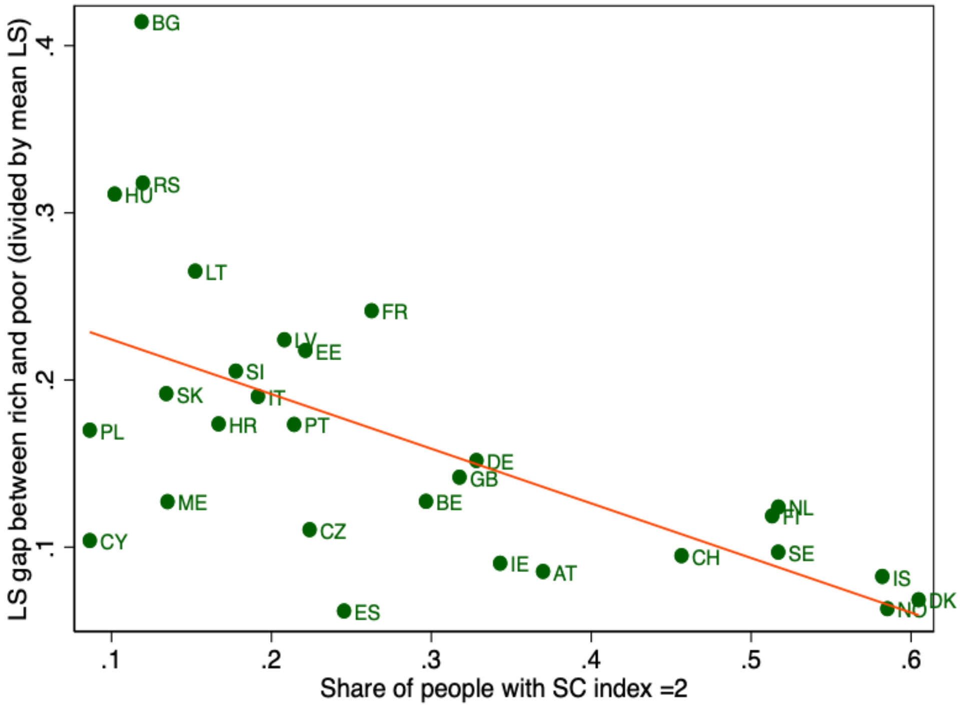

Note: Social capital is measured as the share of respondents with a social capital index = 2.

Table A21: Robustness check of country level analysis on ESS (2018) data using the 90/10 ratio as a measure of income inequality.

	Difference in life satisfaction between rich and poor/ average LS		
	(1)	(2)	(3)
Share of people with SC = 2	-0.248	-0.144	-0.120
	(0.203)	(0.179)	(0.183)
Gini	0.255		
	(0.150)		
GDP per capita (log)	-0.356	-0.608*	-0.599**
	(0.260)	(0.270)	(0.235)
90/10 share4		0.214*	
		(0.0812)	
90/10 cut-off 4			0.209
			(0.168)
N	29	27	27

Standard errors in parentheses

* $p < 0.05$, ** $p < 0.01$, *** $p < 0.001$

Note: The unit of analysis are countries.
Data for Gini and 90/10 ratios are from Eurostat, data for GDP are from the World Bank.
Share refers to share of national equivalised income and cut-off refers to the top cut-off point.
All variables are standardised for comparability.

Table A22: Robustness check of country level analysis on ESS (2018) data using the 50/10 ratio as a measure of income inequality.

	Difference in life satisfaction between rich and poor/ average LS		
	(1)	(2)	(3)
Share of people with SC = 2	-0.248	-0.149	-0.132
	(0.203)	(0.182)	(0.186)
Gini	0.255		
	(0.150)		
GDP per capita (log)	-0.356	-0.621*	-0.619*
	(0.260)	(0.278)	(0.258)
50/10 share		0.194*	
		(0.0747)	
50/10 cut-off			0.165
			(0.163)
N	29	27	27

Standard errors in parentheses

* $p < 0.05$, ** $p < 0.01$, *** $p < 0.001$

Note: The unit of analysis are countries.
Data for Gini and 50/10 ratios are from Eurostat, data for GDP are from the World Bank.
Share refers to share of national equivalised income and cut-off refers to the top cut-off point.
All variables are standardised for comparability.

3 Integrated European Values Study - World Values Study

Table A23: OLS with robust standard errors using WVS-EVS (waves 3-6) data: detailed results.

	(1) Life satisfaction		(2) Life satisfaction	
Social capital index = 1	-0.0636	(0.123)	-0.00946	(0.0404)
Social capital index = 2	0.235*	(0.126)	0.0834**	(0.0424)
Social class (subjective)	-0.381***	(0.0238)	-0.103***	(0.00785)
Social capital index = 1 * Social class (subjective)	0.125***	(0.0282)	0.0277***	(0.00928)
Social capital index = 2 * Social class (subjective)	0.197***	(0.0292)	0.0393***	(0.00983)
Scale of incomes	0.108***	(0.00895)	0.0179***	(0.00299)
Social capital index = 1 * Scale of incomes	-0.0210**	(0.0105)	-0.00383	(0.00347)
Social capital index = 2 * Scale of incomes	-0.0583***	(0.0106)	-0.00968***	(0.00360)
female	0.133***	(0.0172)	0.0538***	(0.00571)
age	-0.0451***	(0.00351)	-0.0168***	(0.00115)
age squared /100	0.0475***	(0.00369)	0.0149***	(0.00121)
Completed (compulsory) elementary education	0.0612	(0.0542)	0.00853	(0.0172)
Incomplete secondary school: technical/vocational type/(Comp	0.151**	(0.0613)	0.0468**	(0.0194)
Complete secondary school: technical/vocational type/Seconda	0.0975*	(0.0546)	0.0332*	(0.0173)
Incomplete secondary: university-preparatory type/Secondary,	0.155***	(0.0595)	0.0265	(0.0188)
Complete secondary: university-preparatory type/Full seconda	0.00783	(0.0559)	0.00657	(0.0179)
Some university without degree/Higher education - lower-leve	-0.0383	(0.0581)	-0.0146	(0.0186)
University with degree/Higher education - upper-level tertia	0.0515	(0.0557)	-0.0117	(0.0178)
x007r==divorced/separated	-0.643***	(0.0343)	-0.225***	(0.0114)
x007r==widowed	-0.471***	(0.0438)	-0.234***	(0.0146)
x007r==single	-0.481***	(0.0281)	-0.180***	(0.00948)
x028r==Part time	-0.0914***	(0.0282)	-0.000732	(0.00947)
x028r==Self employed	-0.0826**	(0.0330)	0.00475	(0.0108)
x028r==Retired	-0.0328	(0.0346)	-0.000170	(0.0114)
x028r==Housewife	0.0246	(0.0328)	0.00837	(0.0105)
x028r==Students	0.0792**	(0.0394)	0.00950	(0.0132)
x028r==Unemployed	-0.583***	(0.0425)	-0.152***	(0.0138)
x028r==Other	-0.318***	(0.0677)	-0.0470**	(0.0218)
one child	-0.0304	(0.0296)	0.0111	(0.00995)
two children	-0.00231	(0.0272)	0.0205**	(0.00924)
three children	0.0523*	(0.0296)	0.0502***	(0.00997)
Constant	8.071***	(0.179)	3.854***	(0.0590)
Controls (socio-demographic, country)	Yes		Yes	
Number of observations	48849		49973	
Adjusted R^2	0.147		0.121	

Standard errors in parentheses. * $p < 0.05$, ** $p < 0.01$, *** $p < 0.001$
Note: Dependent variables: Life satisfaction (1-10). Happiness (1-4).
Omitted categories: "Social capital index = 0", "Social capital index = 0 * household income", "Social capital index = 0 * social class".
Included controls are also country and year dummies.

Table A24: Robustness check using individual dummies of social capital (WVS-EVS, Waves 3-6)

	(1) Life satisfaction	(2) Life satisfaction	(3) Happiness	(4) Happiness
Household income	0.0999***	0.0952***	0.0163***	0.0167***
	(0.00557)	(0.00700)	(0.00183)	(0.00233)
Social class	-0.311***	-0.343***	-0.0902***	-0.0900***
	(0.0146)	(0.0192)	(0.00479)	(0.00630)
Trust in others	0.296***		0.0646*	
	(0.0888)		(0.0297)	
Trust in others*Social class	0.104***		0.0250***	
	(0.0206)		(0.00688)	
Trust in others*Household income	-0.0472***		-0.00702**	
	(0.00732)		(0.00247)	
Putnam's group		-0.0182		0.0385
		(0.0980)		(0.0324)
Putnam's group*social class		0.102***		0.0138
		(0.0227)		(0.00750)
Putnam's group*Household income		-0.0177*		-0.00394
		(0.00815)		(0.00272)
Constant	8.077***	8.088***	3.855***	3.842***
	(0.157)	(0.167)	(0.0513)	(0.0548)
N	49647	49838	50770	50960
Adjusted R^2	0.144	0.139	0.118	0.115

Standard errors in parentheses

* $p < 0.05$, ** $p < 0.01$, *** $p < 0.001$

Note: OLS with robust standard errors. Dependent variables: Life satisfaction (1-10). Happiness (1-4).
Omitted categories: "Social capital index = 0", "Social capital index = 0 * household income", "Social capital index = 0 * social class".
Controls: sex, age, age squared, education, marital status, number of children, labour market status, country and year dummies.

Table A25: Lewbel WVS

	(1) Life Satisfaction
Social Capital index	-0.849
	(0.698)
Social capital index * individual income	-0.0255
	(0.0280)
Social capital index * social class	0.427**
	(0.212)
Social class	-0.713***
	(0.221)
Individual income	0.103***
	(0.0302)
Female	0.124***
	(0.0177)
age	-0.0450***
	(0.00366)
age squared (divided)	0.0474***
	(0.00383)
Completed (compulsory) elementary education	0.0487
	(0.0595)
Incomplete secondary school: technical/vocational type	0.101
	(0.0734)
Complete secondary school: technical/vocational type	0.0507
	(0.0681)
Incomplete secondary: university-preparatory type	0.102
	(0.0731)
Complete secondary: university-preparatory type	-0.0542
	(0.0679)
Some university without degree/Higher education	-0.0867
	(0.0684)
University with degree/Higher education	0.00824
	(0.0640)
divorced/separated	-0.644***
	(0.0347)
widowed	-0.475***
	(0.0441)
single	-0.483***
	(0.0287)
Part-time	-0.103***
	(0.0288)
Self-employed	-0.0984***
	(0.0335)
Retired	-0.0549
	(0.0350)
Housewife	0.0157
	(0.0334)
Student	0.0786*
	(0.0411)
Unemployed	-0.575***
	(0.0496)
Other	-0.336***
	(0.0694)
one child	-0.0325
	(0.0303)
two children	0.00482
	(0.0278)
three children or more	0.0565*
	(0.0303)
Constant	3.441
	(3.104)
Number of observations	48849
Adjusted R^2	0.1293
Hansen Statistic	9.295
p-value	0.1577
First step F test: Social Capital	15.44
First step F test: Social Capital * individual income	126.51
First step F test: Social Capital * reference income	14.48
Kleibergen-Paap Wald F test	11.359
Endogeneity test p-value	0.4179

Standard errors in parentheses

* $p < 0.10$, ** $p < 0.05$, *** $p < 0.01$

Table A26: Correlations table

	social class	household income	SCindex	putnam's group	trust in others
social class	1				
household income	-0.4573*	1			
SCindex	-0.1044*	0.1300*	1		
putnam's group	-0.0758*	0.0849*	0.7628*	1	
trust in others	-0.0694*	0.1011*	0.7009*	0.0734*	1

Table A27: Descriptive statistics (WVS-EVS, Waves 3-6)

variable	obs	mean	sd	min	max
life satisfaction	87177	7.349	1.930	1	10
happiness	86649	3.212	0.640	1	4
female	87113	0.531	0.499	0	1
age	86877	47.32	17.48	15	108
age squared /100	86877	25.44	17.45	2.250	116.6
Inadequately completed elementary education	84189	0.0396	0.195	0	1
Completed (compulsory) elementary education	84189	0.135	0.341	0	1
Incomplete secondary school: technical/vocational type	84189	0.115	0.319	0	1
Complete secondary school: technical/vocational type	84189	0.142	0.349	0	1
Incomplete secondary: university-preparatory type	84189	0.102	0.303	0	1
Complete secondary: university-preparatory type	84189	0.172	0.378	0	1
Some university without degree/Higher education	84189	0.124	0.330	0	1
University with degree/Higher education	84189	0.171	0.377	0	1
married	86659	0.614	0.487	0	1
divorced/separated	86659	0.0841	0.277	0	1
widowed	86659	0.0700	0.255	0	1
single	86659	0.232	0.422	0	1
Full-time	86444	0.408	0.491	0	1
Part-time	86444	0.0941	0.292	0	1
Self-employed	86444	0.0621	0.241	0	1
Retired	86444	0.205	0.404	0	1
Housewife	86444	0.0979	0.297	0	1
Students	86444	0.0551	0.228	0	1
Unemployed	86444	0.0539	0.226	0	1
no child	56256	0.306	0.461	0	1
Other	86444	0.0239	0.153	0	1
one child	56256	0.147	0.354	0	1
two children	56256	0.303	0.460	0	1
three children	56256	0.244	0.429	0	1
Social class (subjective)	53168	3.111	0.897	1	5
Scale of incomes	52912	5.073	2.413	1	10
trust in others	84755	0.406	0.491	0	1
membership in at least 1 Putnam's group	85240	0.575	0.494	0	1
Social capital index (0-2)	82941	0.985	0.751	0	2
Year survey	87177	2006	5.178	1994	2014
Country/region	87177	439.4	255.0	20	909

Table A29: Descriptive statistics: WVS-EVS (waves 3-6), country level data.

	count	mean	sd	min	max
Life satisfaction gap between rich and poor / average LS	60	.1821188	.105506	-.0333353	.4852966
Share of people with SC index = 2	63	.2800295	.1679835	.0134298	.658322
GDP per capita (log)	63	10.64228	.3476524	9.86037	11.59522
Gini	63	31.67036	4.306507	23.25295	44.24099
Year survey	63	-	-	1994	2014

Table A28: Detailed results of the country level analysis using WVS-EVS (waves 3-6) data.

	Difference in life satisfaction between rich and poor / average LS			
	(1)	(2)	(3)	(4)
Share of people with SC index = 2	-0.140*			-0.131
	(0.0542)			(0.0714)
Gini index		0.215		0.00566
		(0.158)		(0.190)
Gdp per capita (log)			-0.467*	-0.0821
			(0.215)	(0.259)
Number of observations	60	60	60	60
Adjusted R^2	0.242	0.133	0.162	0.206

Standard errors in parentheses
* $p < 0.05$, ** $p < 0.01$, *** $p < 0.001$
Note: OLS with robust standard errors. The unit of analysis are countries.
All variables are standardised for comparability.

Table A30: List of developed countries included in the analysis of WVS-EVS (waves 3-6) data.

Andorra	Germany	Malta	United Kingdom
Australia	Greece	Netherlands	United States
Austria	Hong	New	
Belgium	Iceland	Norway	
Canada	Ireland	Portugal	
Taiwan	Italy	Singapore	
Cyprus	Japan	Spain	
Finland	South	Sweden	
France	Luxembourg	Switzerland	

Table A31: Robustness check of country level analysis on WVS data using the 90/10 ratio as a measure of income inequality

	Difference in life satisfaction between rich and poor	
	(1)	(2)
Share of people with SC = 2	-0.131	-0.115
	(0.0714)	(0.104)
Gdp per capita (log)	-0.0821	-0.0525
	(0.259)	(0.430)
Gini	0.00566	
	(0.190)	
90/10		0.0652
		(0.323)
N	60	41

Standard errors in parentheses. * $p < 0.05$, ** $p < 0.01$, *** $p < 0.001$
Note: The unit of analysis are countries.
Data for Gini, 90/10 and for GDP are from the World Bank. Share refers to share of national equivalised income and cut-off refers to the top cut-off point.
All variables are standardised for comparability.

4 German SOEP

Table A32: OLS with robust standard errors and individual fixed effects using SOEP data: detailed results.

	(1) Life Satisfaction	
Social capital index = 1	-0.995	(0.835)
Social capital index = 2	-1.960**	(0.874)
Social capital index = 3	-1.619	(1.041)
Social capital index = 4	-1.933	(1.424)
Log of individual income	0.474***	(0.0413)
Social capital index = 1 * Log of individual income	-0.0824**	(0.0401)
Social capital index = 2 * Log of individual income	-0.127***	(0.0425)
Social capital index = 3 * Log of individual income	-0.207***	(0.0481)
Social capital index = 4 * Log of individual income	-0.248***	(0.0632)
Log of reference income	-0.698***	(0.145)
Social capital index = 1 * Log of reference income	0.246**	(0.115)
Social capital index = 2 * Log of reference income	0.429***	(0.121)
Social capital index = 3 * Log of reference income	0.466***	(0.143)
Social capital index = 4 * Log of reference income	0.550***	(0.199)
Age	-0.0139***	(0.00478)
Age squared (divided by 100)	-0.0106**	(0.00460)
Married	0.132***	(0.0315)
Widowed	-0.131**	(0.0624)
Divorced/Separated	0.0245	(0.0471)
Years of education	-0.0139*	(0.00739)
Unemployed	-0.591***	(0.0287)
Student	0.0813**	(0.0403)
Retired	0.0271	(0.0313)
Non working	-0.0310	(0.0195)
House owner	0.0663***	(0.0208)
East Germany	-0.288**	(0.135)
Disability Status of Individual	-0.289***	(0.0315)
Constant	9.434***	(1.050)
Controls (socio-demographic, region, year)	Yes	
Individual fixed effects	Yes	
Number of observations	129901	
Number of individuals	36599	
R^2 within	0.0390	
R^2 between	0.0636	
R^2 overall	0.0585	

Note: * $p < 0.05$, ** $p < 0.01$, *** $p < 0.001$. Omitted categories: "Social capital index = 0 * log of individual income", "Social capital index = 0 * log of reference income". Controls: regional dummies, year dummies.

We note that the main effect of social capital becomes negative when the estimation includes the interaction term between reference income and social capital.

We do not have an explanation for that, hence we suggest further research on this topic.

Table A33: Robustness check using the single dummies for social capital rather than the index (SOEP).

	(1) Social gathering	(2) Helping friends	(3) Performing volunteer work	(4) Participation in local politics
SC	-1.376**	-0.702	-0.311	-1.470*
	(0.680)	(0.533)	(0.616)	(0.877)
Log of individual income	0.455***	0.408***	0.403***	0.377***
	(0.0362)	(0.0240)	(0.0245)	(0.0223)
SC × Log of individual income	-0.115***	-0.0848***	-0.114***	-0.0610
	(0.0334)	(0.0250)	(0.0272)	(0.0395)
Log of reference income	-0.658***	-0.480***	-0.467***	-0.448***
	(0.131)	(0.112)	(0.113)	(0.109)
SC × Log of reference income	0.331***	0.193***	0.161*	0.259**
	(0.0939)	(0.0744)	(0.0861)	(0.124)
Constant	9.270***	8.475***	8.437***	8.482***
	(0.946)	(0.804)	(0.805)	(0.778)
Controls (socio-demographic, region, year)	Yes	Yes	Yes	Yes
Individual fixed effects	Yes	Yes	Yes	Yes
Number of observations	129901	129901	129901	129901
Number of individuals	36599	36599	36599	36599
R^2 within	0.0388	0.0358	0.0347	0.0344
R^2 between	0.0622	0.0527	0.0502	0.0491
R^2 overall	0.0575	0.0491	0.0471	0.0462

Note: OLS with individual fixed effects and robust standard errors.
Dependent variable: Life satisfaction (0-10). Controls: sex (omitted due to fixed effects), age, age squared, marital status, years of education, labour market status, house owner, disability status of individual, living in East Germany, regional dummies, year dummies.
* $p < 0.05$, ** $p < 0.01$, *** $p < 0.001$ Standard errors in parentheses.

Table A34: Correlations table

	Social capital index	Soc Gath Monthly	Help Fre Monthly	Volunt Monthly	Local participation	Individual income	Reference income
Social capital index	1						
Soc Gath Monthly	0.6045*	1					
Help Fre Monthly	0.6901*	0.3067*	1				
Volunt Monthly	0.6553*	0.1027*	0.1470*	1			
Local participation	0.4886*	0.0295*	0.0745*	0.3701*	1		
Individual income	0.1046*	0.0851*	-0.0273*	0.1326*	0.0888*	1	
Reference income	0.0463*	0.0123*	-0.0297*	0.0987*	0.0443*	0.2464*	1

Table A35: Descriptive statistics (SOEP).

	count	mean	sd	min	max
Life satisfaction	129901	6.918122	1.783707	0	10
Individual income (2011 EUR)	129901	1732.723	1027.361	0	44728.43
Log of individual income	129901	7.341583	.4721802	0	10.70839
Reference income (2011 EUR)	129901	1724.819	249.5303	1192.22	2243.969
Log of reference income	129901	7.443037	.1444213	7.084411	7.716447
Soc Gath Monthly	129901	.7748593	.4176765	0	1
Help Fre Monthly	129901	.4126373	.4923105	0	1
Volunt Monthly	129901	.2890124	.4533054	0	1
Local participation	129901	.090569	.2869963	0	1
Social capital index (0-4)	129901	1.567078	1.0297	0	4
Social capital index = 0	129901	.1552875	.3621799	0	1
Social capital index = 1	129901	.3352784	.4720895	0	1
Social capital index = 2	129901	.335671	.4722263	0	1
Social capital index = 3	129901	.1345948	.3412916	0	1
Social capital index = 4	129901	.0391683	.1939959	0	1
Age	129901	47.77856	17.12055	16	101
Age squared (divided by 100)	129901	25.75902	17.28232	2.56	102.01
Single	129901	.2156334	.4112626	0	1
Married	129901	.6354378	.481309	0	1
Widowed	129901	.0660349	.2483441	0	1
Divorced or separated	129901	.0828939	.2757228	0	1
Years of education	129901	11.81008	2.652272	7	18
Working	129901	.585977	.4925544	0	1
Unemployed	129901	.0557501	.2294395	0	1
Student	129901	.0240568	.1532261	0	1
Retired	129901	.171777	.3771879	0	1
Not working	129901	.1624391	.3688545	0	1
House owner	129901	.4866552	.4998238	0	1
East Germany	129901	.2652328	.4414588	0	1
Baden-Wuerttemberg	129901	.1240175	.3296028	0	1
Bavaria	129901	.1362191	.3430224	0	1
Berlin	129901	.0385986	.1926371	0	1
Brandenburg	129901	.0447341	.2067202	0	1
Bremen	129901	.0069976	.083359	0	1
Hamburg	129901	.0133948	.1149587	0	1
Hesse	129901	.0694452	.2542107	0	1
Mecklenburg-Western Pomeran	129901	.0269513	.1619417	0	1
Lower Saxony	129901	.085288	.2793109	0	1
North Rhine-Westphalia	129901	.1971578	.397854	0	1
Rhineland-Palatinate	129901	.0495454	.2170047	0	1
Saarland	129901	.0063279	.0792963	0	1
Saxony	129901	.079576	.2706367	0	1
Saxony-Anhalt	129901	.0472129	.2120947	0	1
Schleswig-Holstein	129901	.0260275	.1592177	0	1
Thuringia	129901	.0485062	.2148341	0	1
1992	129901	.0884751	.2839857	0	1
1994	129901	.0849339	.2787844	0	1
1996	129901	.0855652	.2797219	0	1
1997	129901	.0838177	.2771153	0	1
1999	129901	.0986982	.2982576	0	1
2005	129901	.1443869	.3514829	0	1
2007	129901	.1440559	.3511477	0	1
2009	129901	.1416309	.3486726	0	1
2011	129901	.1284363	.3345762	0	1
Has disability	129901	.1117389	.3150462	0	1

Table A36: Lewbel: SOEP

	(1)
	LifeSat
Social Capital index	1.099
	(1.745)
Social capital * individual income	-0.812***
	(0.275)
Social capital * reference income	0.656***
	(0.150)
Individual income	1.614***
	(0.416)
Reference income	-1.437***
	(0.255)
Female	0
	(.)
Age	-0.0146***
	(0.00432)
Age Squared	-0.00892**
	(0.00417)
Married	0.113***
	(0.0296)
Widowed	-0.151***
	(0.0554)
Divorced/Separated	0.0207
	(0.0440)
Years of education	-0.0101
	(0.00694)
Unemployed	-0.588***
	(0.0279)
Student	0.0731*
	(0.0396)
Retired	0.0399
	(0.0292)
Non working	-0.0235
	(0.0187)
House Owner	0.0915***
	(0.0214)
East Germany	-0.295**
	(0.130)
Has disability	-0.285***
	(0.0295)
Region and year dummies	Yes
N	119701
Adjusted R^2	-0.0084
Hansen Statistic	1.562
p-value	0.6680
First step F test: Social Capital	36.98
First step F test: Social Capital * individual income	16.29
First step F test: Social Capital * reference income	235.63
Kleibergen-Paap Wald F test	10.627
Endogeneity test p-value	0.0001

Standard errors in parentheses

* $p < 0.10$, ** $p < 0.05$, *** $p < 0.01$

5 Lewbel Method of heteroskedasticity generated instruments

There are various reasons to believe that social capital is endogenous to wellbeing, as the association between social capital, social comparisons and subjective wellbeing may be driven by omitted variables or reverse causality. We account for endogeneity using a Two-Stages Least Squares (2SLS) instrumental variable approach. Specifically, we instrument the main effect of social capital, and its interaction terms with absolute and reference income.

Identifying a proper instrument for social capital is difficult, as most of the factors affecting people's social life will likely affect their wellbeing as well. To overcome this problem we use the method of generated instruments proposed by Lewbel (2012). This approach allows to identify a causal model without imposing the exclusion restriction which is typically required in a standard 2SLS, while instead exploiting the heteroskedasticity of the first step equation to construct the instruments (Lewbel, 2012). As discussed by Lewbel (2012) and Baum and Lewbel (2019), this method is valid when the endogeneity of the instrumented variable comes from an error component that appears in both the reduced form and structural equations. In the case of social capital, the error component may be unobserved individual characteristics, such as personality traits, which affect both subjective wellbeing and social capital.

Formally, we implement the two-stage estimator proposed by Lewbel in the following way: to begin, we regress each endogenous variable on all of the control variables from our main equation of subjective wellbeing $\mathbf{X}$, and the vector of residuals μ_i are retrieved. More specifically, we run the following first-stage regressions:

$$\mathrm{S}ocialCapital_i = \alpha_1 + \theta' \cdot \mathbf{X_i} + \mu_{1,i} \tag{2}$$

$$(SocialCapital * AsboluteIncome)_i = \alpha_1 + \theta' \cdot \mathbf{X_i} + \mu_{2,i} \tag{3}$$

$$(SocialCapital * ReferenceIncome)_i = \alpha_1 + \theta' \cdot \mathbf{X_i} + \mu_{3,i} \tag{4}$$

If the residuals from Eq. 2, 3, 4 are heteroskedastic, instruments can be generated by multiplying them with each of the mean-centered observed variables (X_j), as follows:

$$Z_j = (X_j - \bar{X_j}) \cdot \hat{\mu} \tag{5}$$

where j corresponds to a given control variable from vector $\mathbf{X}$, and $\hat{\mu}$ are the vectors of residuals from Eq. 2, 3 and 4. Hence, for each endogenous variable the number of generated instruments Z is equal to the number of control variables included in the vector $\mathbf{X}$. The predicted values are then used in the second step of the 2SLS framework as follows:

$$SWB_i = \alpha_2 + \pi \cdot Endogen\widehat{ous\ V}ariables_i + \theta \cdot \mathbf{X_i} + \nu_i \tag{6}$$

The Lewbel approach relies on the same assumptions of a standard instrumental variable model, with the addition of two extra conditions. The first is that there exists heteroskedasticity in the first stage equation, that is $Cov(\mathbf{Z}, \mu^2) \neq 0$, where $\mathbf{Z}$ is the vector of instruments constructed from some or all of the variables included in the vector of controls of the structural equation $\mathbf{X}$. The second condition is that there exists a $\mathbf{Z} \subseteq \mathbf{X}$ for which $Cov(\mathbf{Z}, \mu\epsilon) = 0$, where ϵ is the error term of

the structural equation of wellbeing, which would allow the constructed instruments to satisfy the exclusion restriction.

The intuition behind the mechanics of the Lewbel approach comes from a standard linear regression mechanics: the residuals are by construction exogenous to the right hand side variables if the model is correctly specified. This means that if the structural form is correctly specified, the remaining errors are idiosyncratic (Lewbel 2012). Hence, if the chosen $\mathbf{X}$ are exogenous in the structural equation, the instruments created on those $\mathbf{X}$ are also exogenous, and will affect the outcome variable only via the endogenous regressor. As Lewbel and Baum and Lewbel discuss, if this assumption does not hold, that is if $Cov(\mathbf{Z}, \mu\epsilon) \neq 0$, bounds on the causal parameters can still be obtained as long as this covariance is not too large (Lewbel, 2012; Baum and Lewbel, 2019). Lastly, If the residuals are heteroskedastic, they contain information about the the variation of the outcome (endogneous) variable, which makes the instruments relevant.

A plausible cause of heteroskedasticity in equations 2, 3 and 4 may come from the non constant variance in the distribution of the residuals of social capital over the age distribution. We test this with a Breush-Pagan test, which confirms this hypothesis with p-values consistently smaller than 0.001. Additionally, age is exogenously determined with respect to subjective wellbeing, which makes it a valid source to construct the instrument on. Hence, in short, we construct the instruments $\mathbf{Z}$ on demeaned age and age squared, multiplied by the residuals of equations 2, 3 and 4.

5.1 Results: Lewbel moderation effects

Table A37: Moderation effects computed after 2SLS.

	Life Satisfaction SC index increase			
	EU-SILC	ESS	WVS-EVS	SOEP
Absolute income	-38%***	-60% ***	-25%	-50% ***
	(0,043)	(0,083)	(0,195)	(0,04)
Reference income	-56%***			-46%***
	(0,055)			(0,04)
Social class			-60%	
			(0,1201)	
Income rank 1-3		-228,64%		
		(4,016)		
Income rank 8-10		-121%		
		(0,226)		

Note: Moderation effects indicate by how much an increase in social capital reduces the income coefficients of the subjective well-being regression. Method: 2SLS regression with robust standard errors. Standard errors are computed with the error propagation method.

Table A38: Lewbel estimation on other dependent variables

	EU-SILC		WVS	ESS
	FeDep	JobSat	Happiness	Happiness
SC Index	0.246*	0.753	-0.298	7.044***
	(0.138)	(0.952)	(0.228)	(1.296)
SC * Abs Income	0.0612**	-0.154	0.00293	-0.897***
	(0.0273)	(0.120)	(0.00930)	(0.171)
SC * Reference Income	-0.122***	0.121		
	(0.0387)	(0.129)		
SC * Social Class			0.116*	
			(0.0672)	
SC * Income Rank 1-3				-0.567*
				(0.310)
SC * Income Rank 8-10				0.713***
				(0.275)
Absolute Income	-0.193***	0.590***	0.0105	1.137***
	(0.0354)	(0.148)	(0.0100)	(0.166)
Reference Income	0.231***	-0.360*		
	(0.0535)	(0.187)		
Social Class			-0.200***	
			(0.0700)	
Income Rank 1-3				0.426
				(0.316)
Income Rank 8-10				-0.701**
				(0.291)
Constant	1.544***	5.669***	2.131**	-0.565
	(0.216)	(1.201)	(0.980)	(1.220)
Controls	Yes	Yes	Yes	Yes
N	317978	150226	49973	38597
Hansen H test	21.229	1.64	7.473	3.469
P-value	0.0001	0.6502	0.279	0.4826

Note: * $p < 0.05$, ** $p < 0.01$, *** $p < 0.001$, s.e. in parentheses.
Instrumented variables are the “social capital”, “social capital * income” and “social capital * reference income ”.
The method is 2SLS with robust standard errors, where the employed instruments have been generated using the Lewbel method. The social capital variable is treated as continuous to limit the number of instruments necessary for identification.
Controls included in each of the estimated equations are the same those included in the main OLS results.

6 Error Propagation method

We estimate the errors of our moderation effects using the error propagation method.

Formally, the formula for the error propagation method is defined as follows:
let $f(x_1, x_2, ..., x_n)$ be a function which depends on n variables $x_1, ..., x_n$ and the the uncertainty around each variable be defined as $x_i \pm \Delta x_i$, where Δx_i is the error.

If the variables are correlated, the function error Δf is calculated as follows

$$\Delta f = \sqrt{\sum_{i=1}^{n}\sum_{k=1}^{n}(\frac{\delta f}{\delta x_i}\frac{\delta f}{\delta x_k}C_{i,k})}$$

where $C_{i,k}$ is the covariance between the couples of variables, $C_{i,k} = cov(x_i, x_k)$.

In our case, the function is the moderation effect, which is defined as the ratio between the estimated coefficients on the interaction term of social capital with income (reference or absolute), and income. In particular:

$$f = \frac{SC * Income}{Income}$$

where income is either absolute income or reference income, and both terms in the ratio are the estimated coefficients from the equation of well-being on social capital, income and reference income and their interaction (Table 1 in the main document), which we assume are correlated.

After some computation the formula to obtain the errors on the moderation effect can be written as follows:

$$S.E. = \sqrt{\frac{(se_{SC*Inc})^2}{Income^2} + \frac{(SC * Income)^2}{Income^4} \times (se_{Inc}^2) - 2\frac{SC * Income}{(Income)^2}\frac{1}{Income}C_{SC*Inc,Inc}}$$

where se stands for standard error of the incomes and interaction coefficients, and the rest are all estimated coefficients. $C_{SC*Inc,Inc}$ is the covariance between estimated coefficients.
