# Supplementary figures and images for: The moderation effect of social capital in the relationship between own income, social comparisons and subjective well-being: Evidence from four international datasets
Source: PLoS One. 2023 Dec 7;18(12):e0288455. doi: 10.1371/journal.pone.0288455 (PMC10703203; doi:10.1371/journal.pone.0288455)

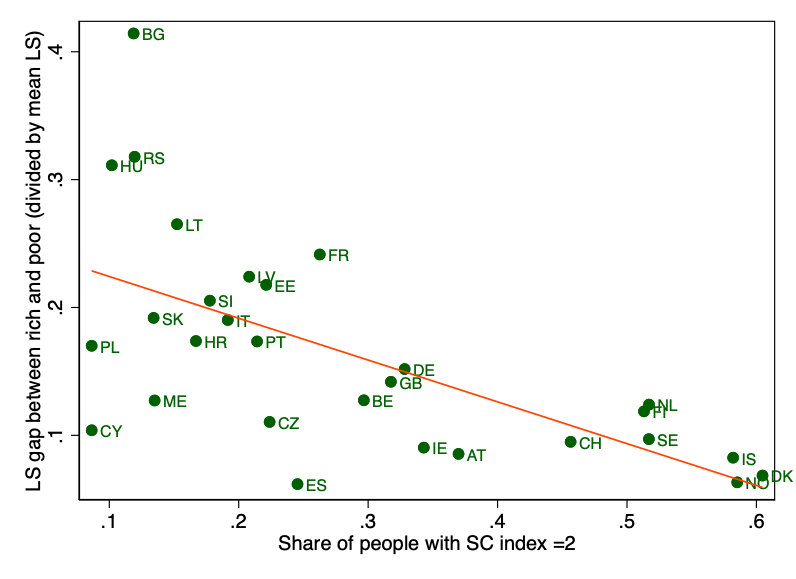

Supplement: S1 File — (ZIP) [file pone.0288455.s002.zip › s1_v03/ESS/FiguraA1b.tif]

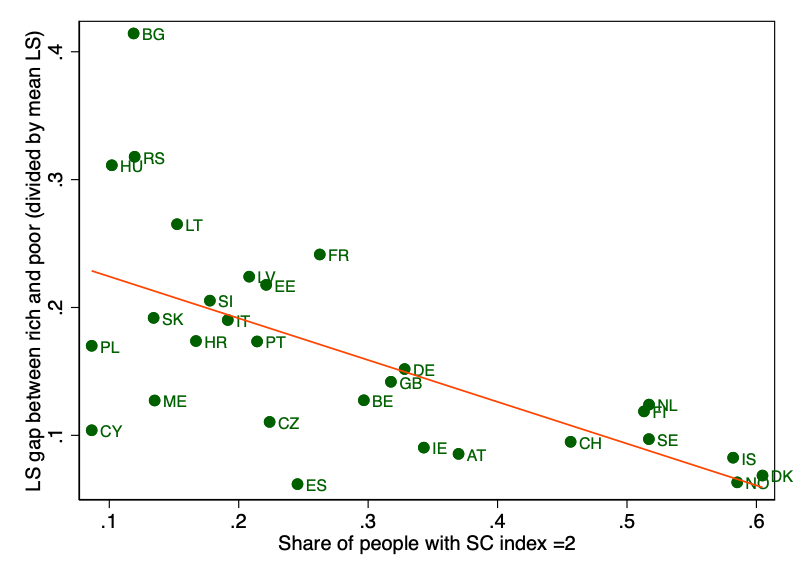

Supplement: S1 File — (ZIP) [file pone.0288455.s002.zip › s1_v03/ESS/FiguraA1b.png]
